# Kinship clustering within an ecologically diverse killer whale metapopulation

**DOI:** 10.1038/s41437-024-00740-y

**Published:** 2025-01-20

**Authors:** Chérine D. Baumgartner, Eve Jourdain, Sebastian Bonhoeffer, Katrine Borgå, Mads P. Heide-Jørgensen, Richard Karoliussen, Jan T. Laine, Aqqalu Rosing-Asvid, Anders Ruus, Sara B. Tavares, Fernando Ugarte, Filipa I. P. Samarra, Andrew D. Foote

**Affiliations:** 1 Department of Environmental Systems Science, Swiss Federal Institute of Technology Zurich, Zurich, Switzerland; 2 Orcestra, Zurich, Switzerland; 3 Norwegian Orca Survey, Andenes, Norway; 4 Section for Aquatic Biology and Toxicology, Department of Biosciences, University of Oslo, Oslo, Norway; 5 Greenland Institute of Natural Resources, Nuuk, Greenland; 6 Department of Natural History, Norwegian University of Science and Technology, Trondheim, Norway; 7 Norwegian Institute for Water Research, Oslo, Norway; 8 Cetacean Research Program, Fisheries and Oceans, Nanaimo, Canada; 9 Westman Islands Research Centre, University of Iceland, Vestmannaeyjar, Iceland; 10 Centre for Ecological and Evolutionary Synthesis, Department of Biosciences, University of Oslo, Oslo, Norway

**Keywords:** Population genetics, Genetic variation, Conservation biology, Population dynamics

## Abstract

Metapopulation dynamics can be shaped by foraging ecology, and thus be sensitive to shifts in prey availability. Genotyping 204 North Atlantic killer whales at 1346 loci, we investigated whether spatio-temporal population structuring is linked to prey type and distribution. Using population-based methods (reflecting evolutionary means), we report a widespread metapopulation connected across ecological groups based upon nuclear genome SNPs, yet spatial structuring based upon mitogenome haplotypes. These contrasting patterns of markers with maternal and biparental inheritance are consistent with matrilineal site fidelity and philopatry, and male-mediated gene flow among demes. Connectivity between fish-eating and ‘mixed-diet’ (eating both fish and mammal prey) killer whales, marks a deviation within a species renowned for its marked structure associated with ecology. However, relatedness estimates suggest distinct spatial clusters, and heterogeneity in recent gene flow between them. The contrasting patterns between inference of structure and inference of relatedness suggest that gene flow has been partially restricted over the past two to three generations (50–70 years). This coincides with the collapse of North Atlantic herring stocks in the late 1960s and the subsequent cessation of their seasonal connectivity. Statistically significant association between diet types and assignment of Icelandic killer whales to relatedness-based clusters indicated limited gene flow was maintained through Icelandic ‘mixed-diet’ whales when herring-mediated connectivity was diminished. Thus, conservation of dietary variation within this metapopulation is critical to ensure genetic health. Our study highlights the role of resource dynamics and foraging ecology in shaping population structure and emphasises the need for transnational management of this widespread migratory species and its prey.

## Introduction

Populations are groups of conspecific individuals whose demographic characteristics, genetic composition and spatial and/or temporal distributions are distinct from one another (Wells and Richmond 1995). Thus, identifying populations is fundamental for ecological studies and targeted conservation strategies. Characterising population structure, defined as the organisation of genetic variation driven by evolutionary processes (Wright 1949), can help define biologically meaningful conservation and management units (Funk et al. 2012; Hohenlohe et al. 2021; Palsbøll et al. 2007).

Genetic drift and migration are fundamental mechanisms of evolutionary change and strong opposing forces influencing the population structure of wild species. Genetic drift is characterised by random fluctuations in allele frequencies and may lead to genetic differentiation in fragmented wild populations (Barton and Whitlock 1997; Wright 1931). In a metapopulation, gene flow due to migration can counteract genetic drift, maintaining some level of genetic diversity across locally breeding subpopulations (demes), dependent upon immigration rates (Barton and Whitlock 1997; Hanski and Gilpin 1991; Hanski and Simberloff 1997).

Marine predators can be wide-ranging and highly selective in their resource and habitat use. Thus, the distribution and abundance of prey can influence their migration routes, site fidelity, and behaviour (Hays et al. 2016). Where the distribution and abundance of food sources have shifted over time, owing to climate change or other anthropogenic pressures, there are new opportunities to study the interplay of prey distribution and metapopulation dynamics in natural ecosystems.

The killer whale (*Orcinus orca*) is a top marine predator with a global distribution (Forney and Wade 2007). Despite a capacity for long-distance dispersal, both geographic separation and ecological specialisation have shown to drive fine-scale population structure in this species (Hoelzel et al. 2007; Morin et al. 2010; Parsons et al. 2013). The best-studied killer whale populations in the North Pacific are prey specialists (*ecotypes*; Ford et al. 1998; Krahn et al. 2007). Partially sympatric ecotypes, targeting either mammal or fish prey, are strictly socially segregated (Bigg et al. 1990; Ford et al. 2000). In addition, different populations of fish-eating killer whales display strong geographic site fidelity linked to predictable prey resources (Bigg 1982; Bigg et al. 1990; Hanson et al. 2021; Saulitis et al. 2000). All this results in highly structured genetic variation at two levels: between ecotypes (Hoelzel et al. 1998) and among populations within ecotypes with mostly distinct ranges (Parsons et al. 2013). However, past studies of some North Atlantic killer whale populations have suggested a contrast with the fine-scale structuring found in the North Pacific (Foote et al. 2013).

In the North Atlantic, killer whales that associate with Atlantic herring (*Clupea harengus*) as their main food source (i.e. off Norway and Iceland; Christensen 1988; Jonsgård and Lyshoel 1970; Jourdain et al. 2020; Remili et al. 2023; Samarra et al. 2017a, 2017b; Sigurjónsson et al. 1988; Similä and Ugarte 1993; Similä et al. 1996) are genetically segregated from neighbouring populations for which there are limited ecological data (Foote et al. 2011). Atlantic herring comprises the world’s largest herring stocks (Dragesund et al. 1997; Hay et al. 2000) supporting several thousand killer whales (Christensen 1988; Jonsgård and Lyshoel 1970; Jourdain et al. 2021; Pike et al. 2020; Samarra et al. 2017a, 2017b; Sigurjónsson et al. 1988; Similä and Ugarte 1993; Similä et al. 1996). Norwegian and Icelandic killer whales associate primarily with two regionally distinct stocks: the Norwegian spring-spawning (NSS) herring (Christensen 1988; Jonsgård and Lyshoel 1970; Similä and Ugarte 1993; Similä et al. 1996) and the Icelandic summer-spawning (ISS) herring (Samarra et al. 2017a; Sigurjónsson et al. 1988), respectively.

**Fig. 1:**
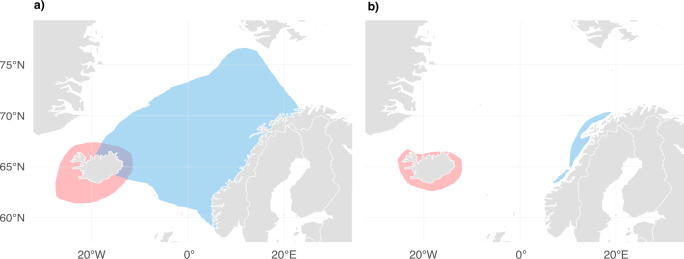
Distribution of North Atlantic herring stocks before and after their collapse in the late 1960s. Distribution of Icelandic summer-spawning (ISS) herring is shown in red and Norwegian spring-spawning (NSS) herring in blue. **a** Distribution of NSS and ISS herring before stock collapse in the late 1960s (adapted from Dragesund et al. 1997; Jakobsson and Østvedt 1999; Røttingen 1990). **b** Distribution after stock collapse, NSS herring data shown for 1974–1986 (adapted from Dragesund et al. 1997) and ISS herring for 1978–1992 (adapted from Jakobsson and Stefànsson 1999; Óskarsson et al. 2009).

The NSS and ISS herring stocks seasonally overlapped northeast of Iceland (Fig. 1a; Dragesund et al. 1997; Jakobsson and Østvedt 1999; Røttingen 1990), before both herring stocks were overfished and collapsed in the late 1960s (Fig. 1b; Dragesund et al. 1997, 1980; Jakobsson 1980; Jakobsson and Stefànsson 1999; Óskarsson et al. 2009). This change discontinued the seasonal overlap of NSS and ISS herring stocks (Jakobsson and Østvedt 1999), representing an abrupt shift in the distribution of the major North Atlantic killer whale prey resource.

Whaling data for the period 1938–1967 suggested a continuous distribution of killer whales across the Northeastern Atlantic (Jonsgård and Lyshoel 1970). However, it is unknown whether this finding was driven by killer whales following herring migrations and overlapping accordingly before the stock collapse. We hypothesise that the historical seasonal overlap in the range of herring stocks facilitated gene flow in herring-associated killer whales, resulting in a connected metapopulation (hypothesis 1). Post-stock collapse, photo-identification data of individual killer whales failed to identify regular movement of known herring-feeding whales between Norwegian and Icelandic herring grounds (Foote et al. 2010). A group of four whales, encountered seven times off Iceland 2018–2024, was observed once off the Norwegian coast in 2022 (Mrusczok et al. 2024), but the individuals have not been previously identified on the Norwegian herring grounds (Jourdain, unpublished data). Thus, we hypothesise that killer whales following the Icelandic and Norwegian herring stocks have become spatially segregated upon the herring stock collapse (hypothesis 2).

While herring is recognised as the main food source in herring-associated killer whales, more complex foraging ecology has been documented in the waters off Iceland and Norway. Notably, dietary diversity is not a recent development due to the herring stock collapse. The first predation records revealing dietary diversity in killer whales off Norway date back to the early 20th century (Christensen 1982; Collett 1912; Foote et al. 2009, 2013; Jonsgård 1968; Jonsgård and Lyshoel 1970). This heterogeneity has been shown to dictate movement patterns of different social groups. Within Icelandic waters, almost half of individuals sighted more than once followed herring year-round (Marchon et al. 2024; Samarra et al. 2017a). A subset of Icelandic herring-associated killer whales seasonally migrates to Scotland to feed on higher trophic level prey (Samarra and Foote 2015; Samarra et al. 2017b; Scullion et al. 2021). Others are only seen in either herring overwintering or spawning grounds, with their complete movement patterns yet to be discovered (Marchon et al. 2024; Samarra et al. 2017a, 2017b). Alternative prey species from various taxa have been documented around Iceland (Samarra et al. 2018). Off the coast of Norway, several social groups of herring-feeding killer whales are known to, seasonally or opportunistically, feed on other prey types including harbour seal (*Phoca vitulina*), grey seal (*Halichoerus grypus*), harbour porpoise (*Phocoena phocoena*) and lumpfish (*Cyclopterus lumpus*), with noticeable influence on seasonal movements for these whales (Bisther and Vongraven 2001; Jourdain et al. 2017, 2019, 2020; Vogel et al. 2024; Vongraven and Bisther 2013). Biochemical markers supported these observations to be reflecting persistent dietary preferences. For example, killer whales with a diet including seals in Norway and Iceland have elevated δ^15^N isotope values (Jourdain et al. 2020; Samarra et al. 2017b), higher pollutant levels (Andvik et al. 2020; Remili et al. 2021), and distinct fatty acid profiles in their blubber (Remili et al. 2023), relative to other killer whales from the same study area for which there was no evidence of feeding on higher trophic level prey. However, despite variation in foraging ecology, studies within the Icelandic (Tavares et al. 2018) and Norwegian (Jourdain et al. 2024) metapopulation demes have found social and genetic connectivity between diet groups. Thus, we hypothesise that shared dietary components facilitate gene flow beyond ecological groups, and further, across the metapopulation (hypothesis 3).

Contrary to Norway and Iceland, movement of killer whales off Greenland has not been clearly linked to herring migrations. Until about 20 years ago, killer whales were irregularly seen in Greenland, reportedly feeding on marine mammals and fish (Heide-Jørgensen 1988). A regime shift due to climate change and the consequential loss of drift- and sea ice has resulted in yearly sighting of killer whales during summer in East Greenland (Heide‐Jørgensen et al. 2022). Direct observations, indigenous knowledge and analysis of biochemical markers show that killer whales in Greenland have a mixed diet of marine mammals (mainly harp seals, *Pagophilus groenlandicus*) and fish (mainly mackerel, *Scomber scombrus*; Heide-Jørgensen 1988; Remili et al. 2023).

Previous studies have reported that killer whales sampled from East Greenland, Iceland, Scotland, and Norway could be assigned to a single population based on microsatellite markers (Foote et al. 2011, 2013). However, at that time, the herring-associated putative metapopulation was predominantly represented by Norwegian individuals, with only few samples available from Iceland and Greenland to assess the fine-scale structuring within this putative metapopulation. Furthermore, data on foraging ecology of sampled individuals was not available to investigate potential drivers of population structure. Finally, the use of microsatellites in previous studies has hindered cross-comparison in subsequent studies and among laboratories, due to the need for calibration of allele equivalence.

In this study, we investigate whether ecological and spatio-temporal factors contribute to population structuring of North Atlantic killer whales using an extensive data set sampled across a broad geographical range from the southwest of Greenland to Norway. We target genome-wide nuclear single nucleotide polymorphisms (SNPs) using a capture protocol and bait oligos, in addition to downstream data processing following Jourdain et al. (2024), allowing additive comparisons between studies. Using analytical methods that account for both historical and contemporary gene flow (Palsbøll 1999; Palsbøll et al. 2010) we assess temporal effects of shifts in prey distribution and migration on mating patterns (addressing hypotheses 1 and 2). Incorporating multi-decadal sighting and predatory records of individual killer whales, we evaluate the role of different, yet overlapping, foraging ecology in population structuring (addressing hypothesis 3). Killer whale social groups are based around high matrilineal philopatry, and populations typically reflect expanded matrilines, thereby consisting of single, or closely related mtDNA haplotypes. This results in strong structuring at mtDNA haplotypes among populations. Gene flow is primarily male-mediated in other killer whale populations, and acts to homogenise genetic variation across populations (Hoelzel et al. 2007). Therefore, we compare variation at maternally inherited mitochondrial (mtDNA), and biparentally inherited nuclear DNA (nuDNA) markers to investigate sex-biased dispersal, and to differentiate site philopatry and physical dispersal from gametic dispersal. Accordingly, we test if maternal philopatry and male-mediated dispersal underpins structure within this putative metapopulation (hypothesis 4).

## Materials and methods

### Data set

A total of 204 (data set 1, Supplementary Table S1) samples of photo-identified killer whales were included in this study. Photo-identification protocols were based on Bigg (1982), details for the protocol in Norway can be found in Jourdain et al. (2021) and for Iceland in Samarra et al. (2017a). Off the coasts of Iceland (*n* = 72, 2013–2022) and Norway (*n* = 106, 2017–2021), skin biopsy samples were collected from the saddle patch region using an ARTS pneumatic darting system or an injection gun (Pneu-Dart Inc) and stainless-steel sterilised biopsy tips following the protocols described in Samarra et al. 2017b and Jourdain et al. 2020, respectively. Eight additional Norwegian samples were collected during the necropsies of one known, and seven unknown individuals that were found stranded and deceased (Norway, *n*_initial_ = 114). Fifteen samples (2012–2021) from individuals taken by subsistence hunters off the coast of Greenland, were provided by the Greenland Institute of Natural Resources (Greenland, *n*_initial_ = 15). Out of the 72 Icelandic samples, DNA of 60 Icelandic samples was extracted in a previous genetic study (Tavares et al. 2018), and DNA of 12 samples was extracted in this study. All Norwegian sequences, and sequences from three additional captive whales with Icelandic origin and known pedigree (Iceland, *n*_initial_ = 75), were published as part of Jourdain et al. (2024) and downloaded from NCBI (https://www.ncbi.nlm.nih.gov/bioproject/PRJNA956724/). Throughout this manuscript, we refer to killer whales sampled in near-shore field sites of three major regions as the Greenlandic, Icelandic, and Norwegian demes.

Dietary categorisation of individually identifiable, naturally marked killer whales off Iceland and Norway was based on over a decade of predation records (Jourdain et al. 2017, 2019; Samarra et al. 2018), sighting histories brought in relation to spatio-temporal occurrence of main prey resources (Jourdain et al. 2021; Samarra et al. 2017a; Samarra and Foote 2015), and biochemical markers such as stable isotope ratios, pollutant levels and fatty acid profiles (Andvik et al. 2020; Jourdain et al. 2020; Samarra et al. 2017b; Remili et al. 2021, 2023). While foraging ecology among these herring-associated killer whales appears complex, we adopted a previously published simplified but biologically meaningful binary categorisation of individuals, into *fish-eating specialists* and individuals with a *mixed diet* (as per Jourdain et al. 2020, 2024; Remili et al. 2021; Samarra et al. 2017b). Specifically, herring-feeding killer whales never observed feeding on marine mammals had been assigned the *fish-eating* specialist group. Individuals known as fish-eaters and observed feeding on mammal-prey at least once, and/or for which elevated nitrogen isotopic ratios (δ^15^N: ^15^N/^14^N) were documented, had been categorised as *mixed diet*. Killer whales off Greenland were categorised as *mixed-diet*, inferred from fatty acid profiles, stomach contents and worn teeth (Foote et al. 2013; Remili et al. 2023; Ugarte and Rosing-Asvid, unpublished data).

### Library building, sequencing and mapping

DNA was extracted from 12 Icelandic and 15 Greenlandic killer whale samples. Libraries were built for 72 Icelandic (including 60 DNA extracts from Tavares et al. 2018) and 15 Greenlandic samples, and a bait-capture approach was used to enrich the library for 1346 biallelic nuclear SNPs, that had been identified in previous work (Foote and Morin 2016; Moura et al. 2014). The enriched captured pool was sequenced using 150-bp paired-end Illumina sequencing. Mitochondrial genome haplotypes were obtained by shotgun-sequencing pre-captured, amplified libraries on an Illumina MiSeq platform, using the v2 reagent kit with 300 sequencing cycles for paired-end sequencing and a read length of 150 base pairs. More detailed laboratory methods are given in the supplemental materials and Jourdain et al. (2024).

Adapters were removed from sequences using *adapterremoval* (version 2.3.1, Lindgreen 2012; Schubert et al. 2016), and mapped to the nuclear reference genome (NCBI, https://ftp.ncbi.nlm.nih.gov/genomes/all/GCA/000/331/955/GCA_000331955.2_Oorc_1.1/) and mitochondrial reference genome (NCBI, NC_023889.1 *Orcinus orca* isolate ENAHN1 mitochondrion, complete genome); using *bwa* (version 0.7.15, Li 2013; Li and Durbin 2009, 2010). Duplicate reads were removed using *samtools* rmdup (version 1.12, Li et al. 2009). We used Analysis of Next Generation Sequencing Data (*ANGSD* version 0.925, Korneliussen et al. 2014) to estimate depth of coverage and generate nuclear allele frequencies and genotype likelihoods. By taking genotype likelihoods as input rather than called genotypes, uncertainty was incorporated for subsequent analyses (Korneliussen et al. 2014). Command lines used in this study can be found in the supplemental material. Nuclear SNP-genotypes were saved in bam format and mitochondrial genomes in fasta format.

### Population-based genetic inference of population structure

Genotype probabilities were estimated in *ANGSD* and used as input to estimate a covariance matrix using the *PCangsd* algorithm (version 0.98, Fumagalli et al. 2013, 2014; Meisner and Albrechtsen 2018). Assignment of individuals to sampling locations and distribution of genetic variation across geographical locations was investigated using Principal Component Analysis (PCA). In *R*, eigenvectors from the covariance matrix were calculated and Principal Components one and two (Supplementary Fig. S2) visualised with *ggplot2* (version 3.4.4, Wickham 2016) in *RStudio* (*R* version 4.3.0, R Core Team 2023).

Secondly, we investigated if geographical sampling locations corresponded to discrete killer whale populations with an individual-based assignment test. Using *NGSadmix*, a maximum likelihood method that bases its inference on genotype probabilities (version 32, Skotte et al. 2013), we inferred the most likely number of ancestral populations (*K*) and individual assignments to those populations, assuming *K* from one to ten. Five runs were performed for each value of *K*, each with different seeds (Supplementary Fig. S3). Genetic ancestry proportions for each individual and per geographic origin were visualised in *ggplot2* (Wickham 2016) in *Rstudio* (R Core Team 2023). Mean ancestry proportions for each combination of geographical location and ancestry unit were calculated using the code provided on GitHub repository of package *mapmixture* (version 1.1.4, Jenkins 2024). We applied *EvalAdmix* (version 0.95, Garcia-Erill and Albrechtsen 2020) to evaluate the fit of modelled admixture proportions inferred by *NGSadmix*, where pairwise correlation of residuals between individuals will be close to 0 in case of a good fit of the data to the admixture model. We applied this for the entire data set (Supplementary Fig. S4a, b; data set 3, Supplementary Table S1) and for a subset of 20 individuals exhibiting homogeneous ancestry (10 from each extreme of the sorted *NGSadmix* barplot; Supplementary Fig. S4c). The correlation of residuals was visualised in *Rstudio* (R Core Team 2023). See Garcia-Erill and Albrechtsen (2020) for the equation used to estimate the correlation of residuals between pairs of individuals.

### Kinship-based estimation of current migration and gene flow

Identifying contemporary migration patterns in marginally diverged putative populations can be limited using population-based methods. Distribution of samples within PCA space and ancestry proportions reflect evolutionary means of historic dispersal patterns (Palsbøll 1999; Palsbøll et al. 2010). Contrarily, kinship-based methods yield estimates for subtle population structuring and contemporary patterns of gene flow. The captured temporal scope of kinship-based methods depends on generation time, dispersal characteristics, and the degree of relatedness targeted. Focusing on distantly related individuals may extend the applicable time frame, however, limited loci restrict discernible relationships to first and second-order relatives, covering only the two to three previous generations (Palsbøll et al. 2010). To investigate recent gene flow, we employed kinship estimates, focusing on higher-order relationships given these constraints.

Accounting for small effective population sizes known in high latitude killer whale populations (Foote et al. 2021), we used a relatedness estimate (*r*_xy_) that was developed for inbred populations. This measure is based on the proportion of homologous alleles shared by two individuals (*x* and *y*) identical-by-descent (IBD) and has an upper bound of one, despite inbreeding (Hedrick and Lacy 2015). We implemented this method in *NGSrelate* (version 0.1, Korneliussen and Moltke 2015). The symmetrised data was converted into a wide-format matrix, using the acast() function from the *reshape2* package (version 1.4.4, Wickham 2007) and transformed into a distance matrix using 1− *r*_xy_ as the metric in *RStudio* (R Core Team 2023). Hierarchical clustering was performed using average linkage clustering (UPGMA) and Euclidean distance with 10,000 bootstrap resamples, and cluster support was assessed via approximately unbiased (AU) *p*-values, implemented in the *pvclust* package (version 2.2-0, Suzuki and Shimodaira 2006). Results were visualised as a dendrogram with significantly supported clusters highlighted (AU *p*-values greater than 0.95), and as a heatmap of relatedness estimates, *r*_xy_, using *pheatmap* (version 1.0.12, Kolde 2018).

We analysed the association between diet types and cluster assignment for Icelandic individuals, as they were the only ones distributed across multiple clusters. A contingency table (Supplementary Fig. S5a) was constructed, and a two-sided Fisher’s exact test (Fisher 1934) was performed using *RStudio* (R Core Team 2023) to determine if there was a statistically significant relationship between diet and genetic clustering. A *p*-value below 0.05 was considered indicative of statistical significance.

A caveat of the *r*_xy_ statistic when applied to a metapopulation is that it is normalised to account for background relatedness for the entire metapopulation. Given that our data set potentially encompasses multiple demes, we evaluated the effect of subsetting the data on background relatedness. We estimated pairwise relatedness for the complete data set (data set 4, Supplementary Table S1) as well as within each deme. Comparing *r*_xy_ estimates for known pedigrees and distribution of estimates (Supplementary Fig. S6a), we find that relatedness estimates among Greenlandic individuals were higher in the pooled data than when estimated from the Greenlandic deme alone. The number of polymorphic sites is lower within the Greenlandic deme than for the total data set (Supplementary Fig. S6b). Thus, the number of sites being considered in the complete data set, including the non-polymorphic sites within the Greenlandic deme, lead to a higher proportion of homologous alleles shared by two individuals from Greenland, and thereby higher relatedness. There was no strong difference of relatedness estimates and number of polymorphic sites between other demes and the pooled data. Therefore, we conclude that the metapopulation estimated *r*_xy_ values are reliable for relative comparisons rather than absolute metrics.

To infer close relatives in our data set based upon absolute relatedness values, we estimated a further set of metrics, implemented in *NGSrelate* (Korneliussen and Moltke 2015). Firstly, we derived maximum likelihood (ML) estimates that two individuals share zero (*r*_*0*_), one (*r*_*1*_), or two (*r*_*2*_) alleles identical-by-descent at each site across the genome (Cotterman 1940; Jacquard 1972). Secondly, we employed a combination of three statistics - *R0*, *R1* and *KING‐robust kinship* (Lee 2003; Manichaikul et al. 2010; Waples et al. 2019): *R0* is a statistic that provides a general test for relatedness between two individuals, similar to a method suggested by Lee (2003), which tests the null hypothesis that a dyad of individuals is genetically unrelated. While this method is not able to further classify pairwise relationships, *KING‐robust kinship* introduced by Manichaikul et al. (2010) can distinguish between different types of close familial relationships, and is robust to population structure; however, it is not applicable if sequencing depth is insufficient for accurate genotype calling and can be sensitive to SNP ascertainment bias (Waples et al. 2019). Waples et al. (2019) defined *R0*, *KING-robust kinship* and *R1*, a new statistic, as ratios of genome-wide identity-by-state genotype combinations of two individuals, without requiring allele frequencies. The distributions of two combinations of these three statistics, *R1*-*R0* and *R1*‐*KING‐robust kinship*, allow to discern close familial relationships robust against ascertainment bias, and applicable to low-sequencing data (Waples et al. 2019). The distribution of *R1* (Supplementary Fig. S6c) and *KING-robust kinship* (data not shown) within each metapopulation deme were similar to the distribution using the complete data set for the same pairs of individuals, thus, this method is less affected by background relatedness. However, both methods by Cotterman (1940) and Waples et al. (2019) assume absence of inbreeding, while *r*_xy_ was developed for inbred populations (Hedrick and Lacy 2015). Considering the different caveats, we decided to use a combination of the methods for subsequent analyses rather than choosing a single approach.

For all the methods above, the comparison of observed and expected statistics can be used theoretically to uniquely distinguish close familial relationships. For example, expected estimates for parent-offspring (P-O) pairs are *R0* = 0, *R1* = 0.5 and *KING-robust kinship* = 0.25. We compared these statistics for 14 known mother-offspring pairs (Jourdain, Samarra, Ugarte and Rosing-Asvid, unpublished data, respectively) to verify if the statistics provided the expected values for P-O pairs. We then conservatively identified further P-O relationships if they fell within the ranges from the known P-O pairs for all metrics (Supplementary Table S2). During this inference, we discovered two Norwegian killer whales exhibited high *R1* estimates to almost all individuals in this study (data not shown). Thus, we removed those two individuals from kinship-based estimation of current migration and gene flow, resulting in *n* = 169 (data set 4, Supplementary Table S1) individuals considered.

In our analysis, we inferred P-O relationships but refrained from inferring lower order relationships. Due to the random nature of recombination, there is significant biological variation in the amount of IBD-sharing between relatives with the same pedigree relationship. Increasing variance in expected statistics, this limits accurate inference of more distant relatives that are genetically differentiated by more recombination events (Waples et al. 2019).

### Characterising mitochondrial haplogroup network and population differentiation based on mtDNA haplogroup frequencies

Mitochondrial genome sequences (16,390 bp) in fasta format were individually copied, aligned and manually inspected for SNPs in *Seaview* and saved in a new fasta file (version 4, Gouy et al. 2010). To account for potential sequencing errors, singletons were ignored and haplogroups (instead of haplotypes) were visualised with *SNPIT* (version 1.2, O’Toole et al. 2023) in *Python* (version 3.6.1, Python Software Foundation 2017). A mitochondrial haplogroup median joining network was constructed in *PopART* with default settings (version 1.7, Leigh and Bryant 2015).

To assess population differentiation based on mitochondrial DNA haplotype frequencies, we estimated φ_st_ from an analysis of molecular variance (AMOVA, Excoffier et al. 1992; Michalakis and Excoffier 1996) using *GenoDive* (version 3.06, Meirmans 2020). The mitochondrial genome was considered a single locus, and each haplotype treated as an allele. Data were converted into *GenoDive* format using *RStudio* (R Core Team 2023). Statistical significance was determined using 1000 permutations, and the significance threshold adjusted for multiple comparisons using Bonferroni correction (Rice 1989).

## Results

### Genotypes and mitochondrial sequences

Nuclear SNP-genotypes and mitochondrial genome sequences were generated for 72 Icelandic and 15 Greenlandic individuals and combined with existing data from three Icelandic and 114 Norwegian individuals (Jourdain et al. 2024). We found that 348 of 1346 investigated nuclear sites were polymorphic in this data set. We excluded two Greenlandic samples from our analyses as we inferred they belong to a different population (Population B, Foote et al. 2011) based upon mitochondrial haplotypes and ongoing whole genome analyses (Baumgartner and Foote, unpublished data). Samples with a low mean depth of coverage of nuclear markers (two Greenlandic, 17 Icelandic, and 12 Norwegian) or mitochondrial genomes (one Greenlandic and two Norwegian) were excluded from respective bioinformatic analyses (Supplementary Table S1). This resulted in *n* = 199 individuals included in analyses with mitochondrial genomes (data set 2) and *n* = 171 individuals included in population-based analyses (data set 3; mean depth of coverage after mapping was 40.75x (SD = 24.80x), Supplementary Fig. S1). We have publicly archived the sequencing data of samples with sufficient coverage generated in this study (11 Greenlandic and 55 Icelandic individuals) in the NCBI database (IcelandicOrcaProject BioProject; NCBI accession: PRJNA1120234). Two Norwegian samples were outliers in a first run of pairwise relatedness estimates, which were removed from subsequent analyses, resulting in *n* = 169 genotypes used with kinship-based methods (data set 4, Table S1). We compared Sanger sequenced mitochondrial control regions from 60 individuals published in Tavares et al. (2018) with our assembled mitogenomes from the same individuals and found complete concordance.

### Principal component analysis

**Fig. 2:**
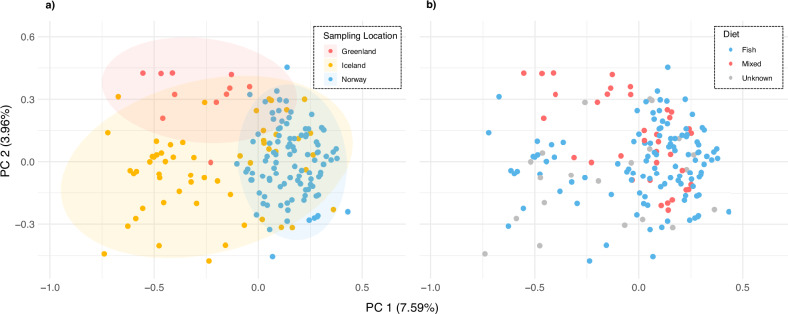
Scatterplots displaying genetic variation along the first two principal components of a Principal Component Analysis (PCA) based on 348 polymorphic SNPs. Genotype likelihoods of *n* = 171 North Atlantic killer whales were used (data set 3). The percentage of variance for each axis is shown in parentheses. **a** Samples are colour-coded by sampling location: Greenland (*n* = 11), Iceland (*n* = 58), and Norway (*n* = 102). Ellipses represent 95% confidence intervals and are colour-coded by the respective sampling locations. **b** Samples are colour-coded according to individual diet: fish-eating specialists in blue (*n*_Norway_ = 85, *n*_Iceland_ = 28) and mixed-diet individuals in red (*n*_Norway_ = 17, *n*_Iceland_ = 8; *n*_Greenland_ = 11). Grey denotes individuals whose diets are partially unknown (*n*_Iceland_ = 22, including three individuals in captivity with Icelandic origin from Jourdain et al. 2024).

No strong spatial clustering in genetic variation was observed in a PCA among samples from Greenland, Iceland and Norway (Fig. 2a). PC1 captured 7.59% of the variance, predominantly driven by the distribution of genetic variation within the Icelandic deme. Greenlandic and Norwegian samples clustered largely within this variation. PC2 explained 3.96% of the variance, driven by outlying Icelandic and Greenlandic samples. No genetic segregation between presumed fish-eating specialists and mixed-diet whales was found in the same PCA, when the data was colour-coded according to individual diet (Fig. 2b). Confidence intervals were omitted given a subset of individuals with unknown diets.

### Individual assignment and admixture analyses

**Fig. 3:**
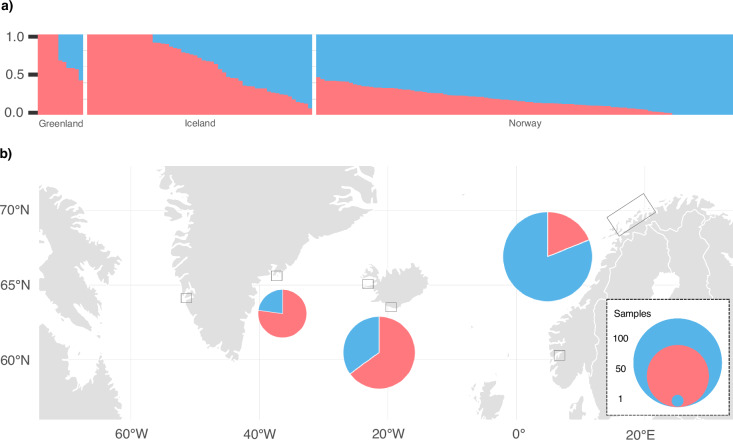
Admixture proportions inferred by *NGSadmix* conditional on two ancestral populations. Ancestry proportions are shown in blue and red. Proportions are based on the highest probability run (of five) assuming two ancestral populations (*K* = 2). The samples (data set 3: *n* = 171) are arranged by sampling origin from West to East; Greenland (Nuuk, *n* = 1; Ammassalik, *n* = 10), Iceland (Kolgrafafjörður and Grundarfjörður, *n* = 21; Vestmannaeyjar, *n* = 34) and Norway (between Andenes and Skjervoy, *n* = 101; Sognefjord, *n* = 1). **a** Each column represents an individual arranged by sampling location and descending proportions for ancestral population one. **b** Mean ancestry proportions are shown as admixture pie charts per geographic origin with circle size representing number of samples.

Admixture inferred by *NGSadmix* yielded the highest probability support for all samples originating from a single population (*K* = 1; Supplementary Fig. S3), consistent with the lack of distinct clusters and low genetic variance between samples within the PCA. The second highest probability support was yielded for two ancestral populations (*K* = 2; Supplementary Fig. S3). Although less well supported, we investigated whether population assignment assuming *K* = 2 provided biologically meaningful clusters. Overall, ancestry proportions inferred by *NGSadmix* assuming *K* = 2 varied West to East (Fig. 3a). Mean ancestry proportions for each sampling location indicated a geographical cline from West to East (Fig. 3b). An evaluation of the admixture model fit using *EvalAdmix* (Garcia-Erill and Albrechtsen 2020) resulted in numerous non-zero correlation of residuals (Supplementary Fig. S4b), suggesting that a cline is not a perfect fit to the data (Garcia-Erill and Albrechtsen 2020).

We found mean correlation of residuals approximating zero between Iceland and Norway, Greenland and Norway and on the population level within Norway (Supplementary Fig. S4b). Positive mean correlations of residuals were observed among individuals within Greenland and within Iceland, likely driven by the inclusion of multiple samples from the same family group. This resulted in negative mean correlations of residuals on the population-level between Iceland and Greenland. For individuals with homogeneous ancestry proportions, our analysis revealed a lack of aberrant patterns in the correlation of residuals (Supplementary Fig. S4c), suggesting an adequate model fit (Garcia-Erill and Albrechtsen 2020).

### Hierarchical clustering of pairwise relatedness estimates and inference of kinship

**Fig. 4:**
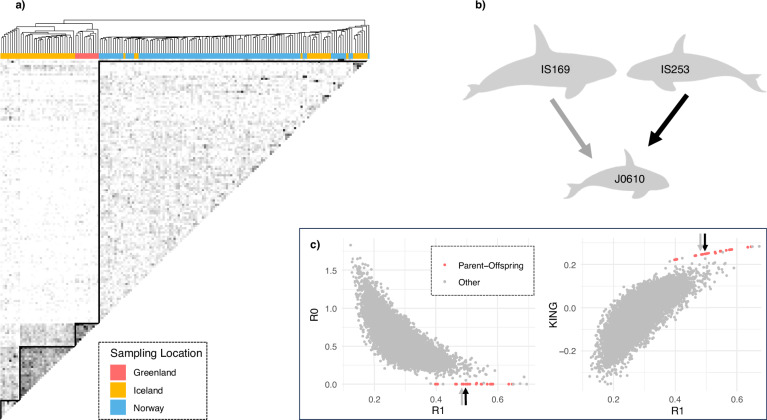
Inference of relatedness based on pairwise relatedness estimates *r*_xy_ and *R0*-*R1* and *KING-R1*. **a** Heatmap and adjacent dendrogram based on hierarchical clustering of pairwise relatedness estimates, *r*_xy_ (data set 4: *n* = 169). Darker shades in the heatmap represent higher relatedness scores, with values ranging from 0 to 1. The diagonal, which depicts pairwise comparisons of each sample with itself, is omitted for clarity. Small subclusters along the diagonal were identified as family groups (data not shown). Individuals in the dendrogram are coloured according to their sampling location. **b** Schematic of a parent-offspring-trio (Supplementary Table S2), consisting of a juvenile with parents that have different dietary preferences (IS169 is a piscivorous male, IS253 is a female with a mixed diet). **c** Scatterplots of two combinations of relatedness metrics: *R0*-*R1* and *KING*-*R1*. Previously known (*n* = 14) and inferred (*n* = 9) parent-offspring relationships are coloured in red. Inferred parent offspring relationships (*n* = 9) were only found within metapopulation demes (Supplementary Table S2). The two parent-offspring relationships in the trio are highlighted using black (IS253-J0610) and grey (IS169-J0610) arrows.

Hierarchical clustering of pairwise relatedness estimates identified 18 significantly supported clusters (AU *p*-value > 0.95, Supplementary Fig. S5a), two of which were dyads. There were four significant spatial clusters rooted in the deeper nodes of the dendrogram: a Greenlandic cluster, two Icelandic clusters, and a cluster that contained Norwegian and Icelandic individuals (red solid squares in Supplementary Fig. S5a). Not all individuals were contained within those clusters: three Icelandic individuals appear between clusters, and a Norwegian killer whale at the edge of the dendrogram. Overall, while Greenlandic and Norwegian individuals formed separate clusters (Supplementary Fig. S5a), Icelandic kin-based groups were more dispersed across the dendrogram (colour-coding according to sampling location, Fig. 4a; Supplementary Fig. S5a).

The same four clusters could be heuristically identified in a heatmap of pairwise relatedness estimates (solid black triangles, Fig. 4a), shaded based upon the relatedness between pairs of individuals. Darker shades indicated recent gene flow between individuals and demes. Notably, only those Icelandic individuals clustering within the Norwegian deme, exhibited gene flow with all clusters, while other clusters in this metapopulation appeared only partially interconnected (shading variation in heatmap, Fig. 4a).

A Fisher’s exact test revealed a statistically significant association between diet types and cluster assignment of Icelandic killer whales (*p* = 0.00964, Supplementary Fig. S5a). Despite no complete segregation of Icelandic diet types, the ecological clustering appeared to be driven by a predominantly piscivorous Icelandic cluster that related with the mixed diet Greenlandic cluster and other Icelandic individuals, but not with Norwegian individuals (I-G, Supplementary Fig. S5a). Icelandic mixed-diet whales were found in the other two clusters (I-I and I-N, Supplementary Fig. S5a), exhibiting recent gene flow with all clusters and diet types.

Thus, clustering in the heatmap and dendrogram reflects kin-based social groups, recent mating between ecological groups, and heterogeneity in recent gene flow between demes.

To further illustrate gene flow across ecological groups and its effects on clustering, we provide an example in the following vignette (Fig. 4b): Icelandic juvenile J0610 has parents with different dietary preferences: IS253 was inferred to be the mother of J0610 based on the following relatedness estimates *r*_xy_ = 0.54, *r*_*0*_ = 0.00, *r*_*1*_ = 0.88, *r*_*2*_ = 0.02, *theta* = 0.28, *R0* = 0.00, *R1* = 0.50, *KING-robust kinship* = 0.25 (Fig. 4c, Supplementary Table S2). This inference was further supported by the constant social association between J0610 and IS253, as documented through photo-identification (Samarra, unpublished data). IS253 is a mixed-diet whale who only partially associates with the herring migration (Samarra et al. 2017b). Her isotopic niche and pollutant levels suggest a diet that includes marine mammals (Remili et al. 2021; Samarra et al. 2017b). The genetically inferred father (IS169) of J0610 (*r*_xy_ = 0.51, *r*_*0*_ = 0.00, *r*_*1*_ = 0.95, *r*_*2*_ = 0.00, *theta* = 0.26, *R0* = 0.00, *R1* = 0.49, *KING-robust kinship* = 0.25), is known to follow herring year-round in Iceland (Samarra et al. 2017b), his isotopic niche and pollutant levels suggest a fish-specialist diet (Remili et al. 2021; Samarra et al. 2017b).

We found that these three individuals clustered together in the dendrogram (within cluster I-I, Supplementary Fig. S5a). To investigate if the high relatedness between IS169 and J0610 influences the association between diet types and cluster assignment of Icelandic killer whales, we removed IS169 from the data set and reran the hierarchical clustering. We found two (instead of four) significant clusters formed in the deeper nodes (Supplementary Fig. S5b); one comprising Greenlandic and Icelandic individuals (western cluster) and one comprising Norwegian and Icelandic individuals (eastern cluster). Fisher’s exact test revealed a statistically significant association between diet types and cluster assignment of Icelandic killer whales (*p* = 0.00170). All Icelandic mixed-diet whales were clustering within the eastern cluster together with Norwegian individuals.

### Mitochondrial haplogroup network and mtDNA-based population differentiation

**Fig. 5:**
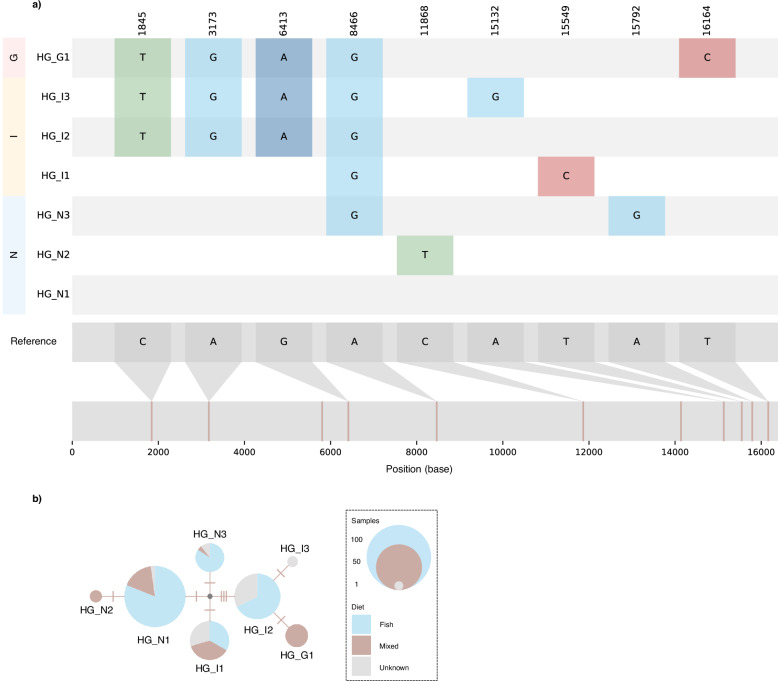
Mitochondrial haplogroups and haplotype-network based on complete mitochondrial genomes. **a** The reference genome is shown at the bottom. The position of nine identified diagnostic SNPs are shown in the first row at the top and are coloured in the following rows according to derived bases (T = green, A = blue, G = turquoise, C = red) respective to the reference genome. It is important to note that real ancestral and derived states are currently unknown. Rows are ordered according to sampling locations and named after the inferred haplogroups; one in Greenland (HG_G1, *n* = 12), three in Norway (HG_N1, *n* = 90; HG_N2, *n* = 3; HG_N3, *n* = 19) and three in Iceland (HG_I1, *n* = 30; HG_I2, *n* = 43; HG_I3, *n* = 2). **b** Haplogroup network with circle size representing number of individuals (data set 2: *n* = 199). Number of SNPs between haplogroups are indicated by tick marks. Pie charts show the proportions of individuals with presumed fish (light blue), mixed (brown) or partially unknown (grey) diets for each haplogroup.

From an alignment of 199 complete mitochondrial genomes, we identified nine diagnostic SNPs which segregated samples into seven mitochondrial haplogroups (Fig. 5a). Each haplogroup exclusively comprised individuals from a single location. *AMOVA* results showed significant differentiation between the three metapopulation demes (Supplementary Table S3). In contrast, no complete ecological separation was found in mitochondrial haplogroups (Fig. 5b).

## Discussion

Understanding genetic connectivity and differentiation among demes is essential to define biologically meaningful conservation units (Funk et al. 2012; Hohenlohe et al. 2021; Palsbøll et al. 2007). Population-based analyses of genotypes at nuclear genome-wide SNPs indicate a connected North Atlantic killer whale metapopulation ranging from the southwest of Greenland to Norway, and across ecological groups. In contrast, maternally inherited mitochondrial genotypes and kinship-based methods reveal distinct clustering within a broader metapopulation framework.

Maternally inherited mitochondrial genome haplogroups were exclusive to either Greenlandic, Icelandic or Norwegian samples within this data set. The statistically significant structuring of mtDNA haplotype frequencies suggests geographic philopatry, a trait broadly found across most killer whale populations studied to date (Morin et al. 2010, 2015). The contrasting patterns of mitochondrial and nuclear markers with maternal and biparental inheritance respectively, are consistent with matrilineal site fidelity and philopatry, and sporadic male-mediated gene flow connecting demes. This has implications for conservation, as the management units should be the smaller and more vulnerable matrilineal demes, rather than the larger metapopulation.

Although this metapopulation is highly interconnected, we identified deviations from random mating. We observe a longitudinal cline of ancestry variation in both mitochondrial haplotype and nuclear genotype allele frequencies. This pattern may be driven by a series of founding events during post-glacial range expansion (Foote et al. 2021; Morin et al. 2015; Filatova et al. 2018), and/or isolation by distance. The latter occurs when individuals from geographically distant populations are less likely to mate, resulting in some level of genetic differentiation over generations (Rousset 1997). Methods based upon evolutionary means (e.g. admixture and PCA analyses) are unable to distinguish between these alternative scenarios. However, kinship-based methods revealed partially restricted gene flow between geographically distant locations in the last two to three generations, consistent with recent isolation by distance.

Historical seasonal overlaps of herring-prey over large geographical scales (Dragesund et al. 1997; Jakobsson and Østvedt 1999; Jakobsson and Stefànsson 1999; Óskarsson et al. 2009; Røttingen 1990) likely provided temporal contact zones and facilitated gene flow among killer whales of the Northeast Atlantic. Our findings of high connectivity based upon evolutionary means are consistent with the hypothesis that killer whales from Norway followed large-scale herring migrations all the way to the northeast of Iceland and overlapped with local killer whale populations before the 1960s herring stock collapse (Foote et al. 2011).

However, periods in which herring stocks are spatially separated may reduce these opportunities for contact, providing windows for genetic differentiation among demes to accrue. Consistent with this, our results based upon relatedness indicate distinct spatial clusters, and heterogeneity in recent gene flow between them. The contrasting patterns of analyses reflecting evolutionary means and recent gene flow, suggest that gene flow among herring-associated killer whales has been partially restricted over the past two to three generations (50–70 years). This coincides with the collapse of North Atlantic herring stocks during the late 1960s and the subsequent cessation of seasonal connectivity between the Norwegian spring-spawning herring and the Icelandic summer-spawning herring. The disruption of an intricate large-scale mating system through habitat/ecological fragmentation can decrease metapopulation connectivity and thus reduce genetic variability (Barton and Whitlock 1997; Wright 1931).

Icelandic kin-based groups were dispersed across the relatedness-based dendrogram, while Greenlandic and Norwegian individuals formed separate clusters. A Fisher’s exact test revealed a statistically significant association between diet types and cluster assignment of Icelandic killer whales. Variation in foraging ecology has been shown to dictate movement patterns of different social groups in Iceland (Marchon et al. 2024; Samarra et al. 2017a), which may underlie this finding. Icelandic fish-eating specialists are thought to follow herring year-round (Samarra et al. 2017b). Icelandic individuals with mixed- or partially unknown diets may not follow herring year-round and a subset seasonally migrates to Scotland (Samarra et al. 2017b; Samarra and Foote 2015). Our results suggest that individuals with a mixed diet can maintain some level of connectivity between Iceland and Norway, although how this connectivity is maintained is unknown. In addition, there may be geographically intermediate unsampled populations that further contribute to this connectivity (Jourdain et al. 2021; Marchon et al. 2024). This role as conduits of gene flow may become more important for connectivity between demes when herring-mediated connectivity is diminished, but this remains untested. Regardless of the mode, this gene flow may retain genetic diversity across the metapopulation to some extent, which can be important for the genetic stability and evolutionary potential of a species in a changing environment (e.g. Hohenlohe et al. 2021).

Prey specialisation promotes strong differentiation between populations of the North Pacific killer whale ecotypes (e.g. Hoelzel et al. 2007; Parsons et al. 2013; Morin et al. 2024). In contrast, we find that fish-eating specialists did not genetically segregate from individuals known to adopt a mixed diet of fish and mammal prey, consistent with previous regional investigations (Foote et al. 2013; Jourdain et al. 2024; Tavares et al. 2018). Through our broad and dense geographic sampling, we were able to detect and report the first confirmed examples of first generation (F1) offspring whose parents had different dietary preferences. We suggest that the connectivity between ecological groups within this North Atlantic metapopulation is in large part resulting from sharing herring as a dietary component. This common prey resource brings killer whale groups together periodically and creates opportunities to mate, regardless of foraging ecology (fish-eating specialists or mixed-diet individuals). Other shared prey resources, for example seals or mackerel (Luque et al. 2006; Nøttestad et al. 2014), may further promote connectivity between Greenland, Iceland and Norway, and merit further investigation.

## Conclusion

This study characterises how decadal changes in the migration patterns of Atlantic herring stocks may have shaped the genetic structure of herring-associated killer whale populations in the North Atlantic. Our study highlights the crucial role of resource dynamics and foraging ecology in shaping population structure, and the importance of considering large-scale ecological factors as well as their spatio-temporal variation in understanding population connectivity in marine predators. This large killer whale metapopulation has historically been subject to large-scale exploitation from commercial whaling and ongoing subsistence hunting, culling to protect herring stocks, entanglement in fishing gear, live captures for the aquarium industry, and faces further threats from contaminant loads, prey resource fluctuations and climate change (reviewed in Jourdain et al. 2019). This study emphasises the necessity for coordinated management of this widespread migratory predator and its prey across national borders to mitigate the risks posed by the threats, and the need for targeted management for the matrilineal demes.

## Supplementary information

Supplemental material

## Data Availability

Raw sequence reads are available at the National Center for Biotechnology Information (NCDBI) under the accession number PRJNA1120234.
