# Supplementary material for: Kinship clustering within an ecologically diverse killer whale metapopulation
Source: Heredity (Edinb). 2025 Jan 20;134(2):109–19. doi: 10.1038/s41437-024-00740-y (PMC11799346; doi:10.1038/s41437-024-00740-y)
Supplement: Supplementary file 1 — Supplemental material [file 41437_2024_740_MOESM1_ESM.pdf]

Supplemental material

Kinship clustering within an ecologically diverse killer whale metapopulation

Chérine D. Baumgartner, Eve Jourdain, Sebastian Bonhoeffer, Katrine Borgå, Mads P. Heide-Jørgensen, Richard Karoliussen, Jan T. Laine, Aqqalu Rosing-Asvid, Anders Ruus, Sara B. Tavares, Fernando Ugarte, Filipa I. P. Samarra, Andrew D. Foote

1. Laboratory methods for DNA extraction, library build and target enrichment capture

In this study, 72 Icelandic and 15 Greenlandic samples were genotyped at 1,346 unlinked genome-wide single nucleotide polymorphisms (SNPs), previously discovered in a global data set (Moura et al. 2014) and filtered for linkage-disequilibrium (Foote and Morin, 2016).

First, DNA was extracted from 12 Icelandic and 15 Greenlandic killer whale samples, using DNeasy Blood and Tissue Kit and the Spin-Column Protocol (Qiagen, Valencia, CA) following the manufacturer's instructions. These 31 DNA extracts and additional 60 Icelandic extracts (Tavares et al. 2018) were fragmented to an average fragment size of approximately 500 base pairs using a Covaris ME220 sonication device. Libraries were built on 72 Icelandic and 15 Greenlandic samples with the *NebNext Ultra II DNA Library Prep Kit for Illumina* (New England Biolabs Inc.) and dual-indexed using NEBNext dual-indexing primer pairs, in the same laboratory and following the same protocol as for the samples in Jourdain et al. (2024). Libraries were pooled equimolar based on amplified library molarity, estimated using an Agilent TapeStation (Agilent Technologies). TapeStation measurements were made of DNA extract prior to and post library assembly. Custom-designed capture using genome-wide biotinylated RNA baits (see Enk et al. 2014) that were manufactured by myBaits Daicel Arbor Biosciences (Design ID: D10110Orca, Reference number: 210810-901, Jourdain et al. 2024) were used to enrich DNA libraries. Both data sets - this study and Jourdain et al. (2024) - were sequenced on a partial lane of an Illumina NovaSeq 6000 sequencing platform.

2. Supplemental tables and figures

Table S1. Sample origin and composition of data sets used for the different analyses.
The Icelandic Orca Project provided 72 samples. Out of the 72 Icelandic samples, DNA of 60 Icelandic samples was extracted in a previous genetic study (Tavares et al. 2018), and DNA of 12 samples was extracted in this study. SNP genotypes and mitochondrial genomes from three captive individuals with Icelandic origin were published in Jourdain et al. (2024) and downloaded from NCBI (https://www.ncbi.nlm.nih.gov/bioproject/PRJNA956724/), resulting in 75 samples with Icelandic origin initially included in this study. One hundred and sixteen genotypes with Norwegian origin were published in Jourdain et al. (2024) and downloaded from NCBI (https://www.ncbi.nlm.nih.gov/bioproject/PRJNA956724/). Two samples were removed because they exhibited not enough coverage in both nuDNA and mtDNA resulting in 114 samples with Norwegian origin initially included in this study. The Greenlandic Institute of Natural Resources provided 15 samples with Greenlandic origin. This sums up to a total of 204 samples initially included in this study (data set 1). For the mtDNA analyses two Greenlandic individuals were removed, as we inferred they belong to a different population (results section *3.1 Genotypes and mitochondrial sequences*). One more Greenlandic and two Norwegian samples were removed due to low coverage, resulting in a total number of 199 samples included in mtDNA analyses (data set 2). Those three samples exhibited high enough coverage in the nuDNA but 17 Icelandic, two Greenlandic, and 12 Norwegian samples were removed because the mean depth of coverage was too low, resulting in 171 genotypes used for population-based methods (data set 3). Two Norwegian samples were outliers in a first run of relatedness estimates, which were removed from subsequent analyses with 169 genotypes using kinship-based methods (data set 4).

**Sample origin**	**Iceland**	**Greenland**	**Norway**	**Total**
Icelandic Orca Project	72			
NCBI BioProject PRJNA956724 (Jourdain et al. 2024)	3		116	
Greenlandic Institute of Natural Resources		15		
Data set 1	75	15	114	**204**
Data set 2	75	12	112	**199**
Data set 3	58	11	102	**171**
Data set 4	58	11	100	**169**

Table S2. ***NGSrelate*** **output for observed and inferred parent-offspring pairs.**
The table organises individuals by geographic origin and includes their IDs, maximum likelihood estimates of genetic relatedness (r_0, r_1, r_2; Jacquard, 1972), pairwise relatedness (r_{xy}; Hedrick and Lacy, 2015), theta (Jacquard, 1972), and R0, R1 and KING kinship coefficients (Lee 2003; Manichaikul et al. 2010; Waples et al. 2019). In the last column relationships are characterised as *P-O*, *high* or *NA* based on association patterns and group affiliations (Jourdain and Samarra, unpublished data, respectively). Observed P-O are based on the constant social association between a female and a calf, as documented through photo-identification (Samarra and Jourdain unpublished data).

Origin	a	b	r0	r1	r2	rxy	theta	R0	R1	KING	Observed association
Greenland	G1	F1	0.0000	0.5684	0.1516	0.6483	0.3699	0.0126	0.5312	0.2519	P-O
	KUL1	K1F	0.0000	0.6825	0.1231	0.6243	0.3577	0.0000	0.5757	0.2676	P-O
	G4	F4	0.0000	0.6127	0.1261	0.6358	0.3618	0.0000	0.4968	0.2492	P-O
Iceland	IS153	IS086	0.0000	0.8581	0.1029	0.5612	0.2855	0.0000	0.5769	0.2679	P-O
	IS253	IS256	0.0000	0.8484	0.0661	0.5544	0.2879	0.0000	0.6354	0.2798	P-O
	IS253	J0610	0.0000	0.8762	0.0166	0.5351	0.2810	0.0000	0.4961	0.2490	P-O
	MAKAIO	KATINA	0.0000	1.0000	0.0000	0.5000	0.2500	0.0000	0.3978	0.2215	P-O
	IS038	IS266	0.0000	0.7957	0.0869	0.5728	0.3011	0.0000	0.5093	0.2523	NA
	IS069	IS008	0.0000	0.9325	0.0517	0.5338	0.2748	0.0000	0.5839	0.2693	high
	IS069	IS046	0.0000	0.9499	0.0001	0.5171	0.2716	0.0000	0.4839	0.2459	high
	IS152	IS169	0.0000	0.8627	0.0523	0.5504	0.2902	0.0000	0.5300	0.2573	NA
	IS172	IS086	0.0000	0.9762	0.0238	0.5119	0.2560	0.0000	0.5480	0.2614	NA
	IS230	IS125	0.0000	0.8855	0.0195	0.5335	0.2786	0.0000	0.5026	0.2507	NA
	J0610	IS169	0.0000	0.9487	0.0000	0.5128	0.2628	0.0000	0.4886	0.2471	NA
Norway	K1	K3	0.0000	0.9734	0.0266	0.5133	0.2567	0.0000	0.6464	0.2819	P-O
	KI01	KI03	0.0000	0.8167	0.1239	0.5792	0.3005	0.0000	0.5808	0.2687	P-O
	KI03	KI06	0.0000	0.8461	0.0725	0.5566	0.2885	0.0000	0.5459	0.2610	P-O
	KI03	KI07	0.0000	0.9154	0.0000	0.5212	0.2712	0.0000	0.4029	0.2231	P-O
	NKW616	NKW616a	0.0000	0.9070	0.0004	0.5330	0.2925	0.0000	0.4629	0.2404	P-O
	NKW785	NKW785a	0.0000	0.9734	0.0000	0.5067	0.2566	0.0000	0.4965	0.2491	P-O
	NKW785	NKW903	0.0000	0.8851	0.0000	0.5287	0.2787	0.0000	0.4048	0.2237	P-O
	NKW1055	NKW1295	0.0000	0.9867	0.0000	0.5033	0.2533	0.0000	0.4661	0.2412	NA
	NKW714	Y137	0.0000	0.7008	0.1359	0.6088	0.3248	0.0000	0.5656	0.2654	NA

Table S3. Population differentiation based on mtDNA haplogroups. The mitogenome was considered a single locus and each haplotype treated as an allele. Shown are φ_{st} values above the diagonal, and p-values below the diagonal, estimated with AMOVA (Excoffier et al. 1992; Michalakis and Excoffier 1996) using GenoDive (Meirmans 2020). The adjusted significance threshold using Bonferroni correction for multiple comparison was α=0.016.

	Iceland	Greenland	Norway
Iceland	-	0.616	0.589
Greenland	0.001	-	0.734
Norway	0.001	0.001	-

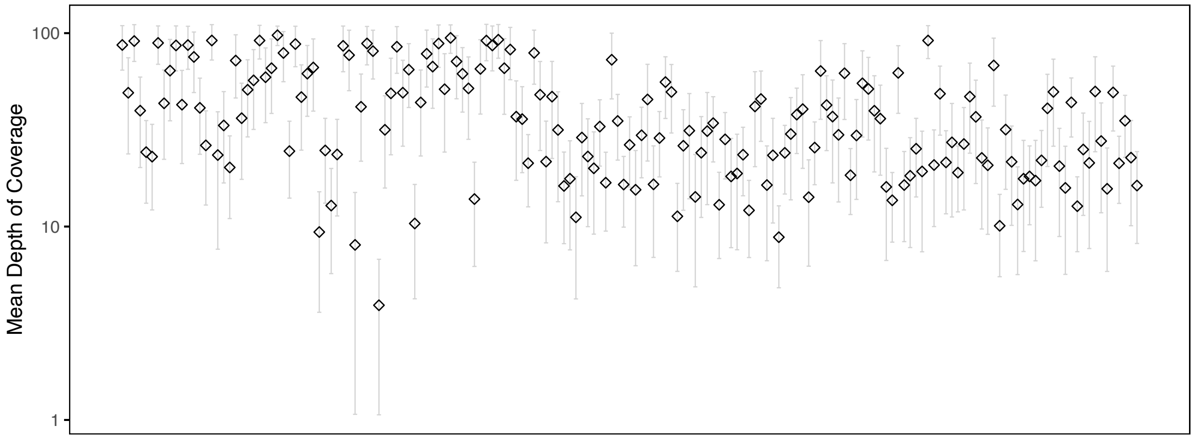

Fig. S1. Mean depth of coverage and standard deviation on the logarithmic scale for each of the sequenced killer whales included in this study (data set 3, n=171). These include uniquely sampled individuals off Greenland (n=11) and Iceland (n=55) sequenced in this study, as well as Icelandic individuals in captivity (n=3) and Norwegian individuals (n=102) that were previously published in Jourdain et al. (2024). The overall mean depth of coverage after mapping was 40.75x (SD = 24.80x), with a range of 3.92x to 97.51x.

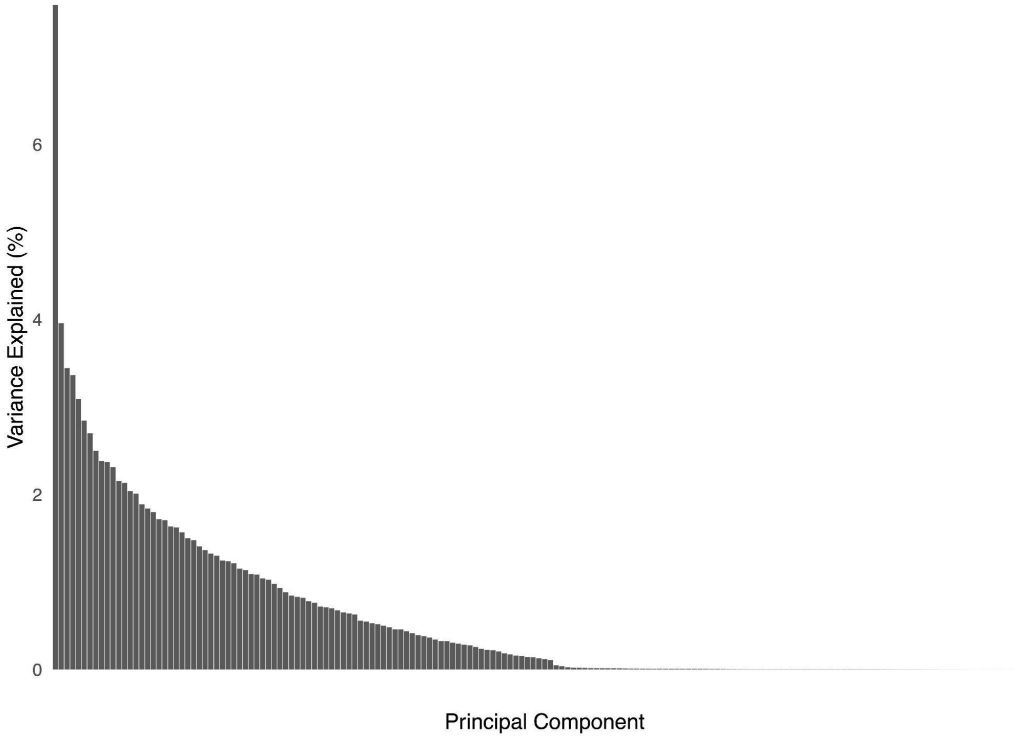

Fig. S2. Principal Components and variance explained (%) of a Principal Component Analysis (PCA).

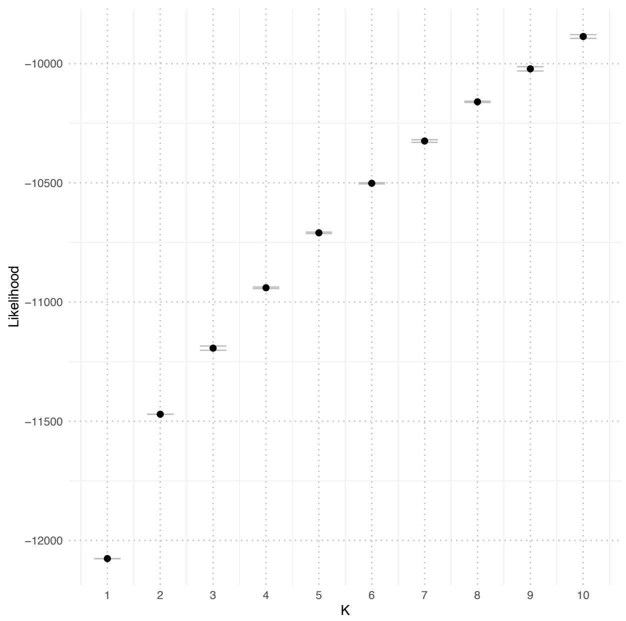

Fig. S3. Likelihood values obtained from *NGSadmix* analysis for different values of K. Likelihood values represent the goodness of fit of the model to the observed data, with lower (more negative) values indicating better fit. For each K (1 to 10), five independent runs were conducted, and the highest log likelihood score from each run was recorded. The figure presents the mean of these highest log likelihood scores along with their standard deviations for each K.

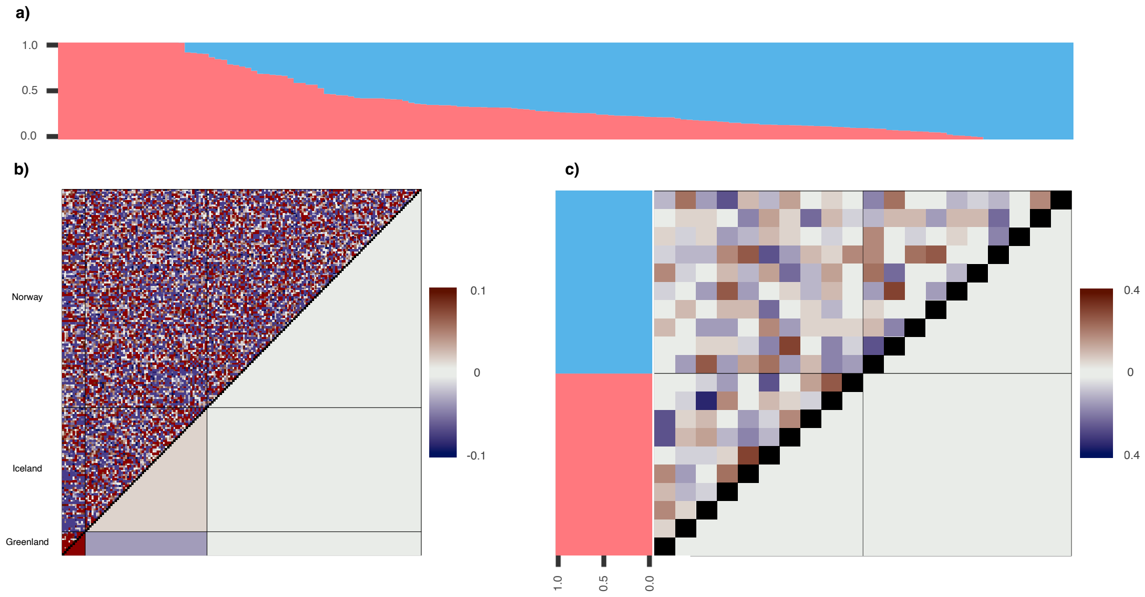

Fig. S4. Evaluation of *NGSadmix*-model fit (K=2) using *EvalAdmix.* **a** *NGSadmix* results for the entire dataset (referenced in Fig. 3), re-sorted by declining proportions for ancestral population one. Due to the nature of the dataset, demes are not explicitly labelled, as they do not exhibit clear segregation. **b** Correlation of residuals for the entire dataset, ordered by metapopulation demes. Individual pairwise correlation of residuals is shown above the diagonal, and mean population correlation of residuals below, ranging from -0.1 to 0.1. **c** Correlation of residuals for a subset of 20 individuals, which exhibited homogeneous ancestry at the extreme ends of the sorted NGSadmix plot (horizontal barplot), ranging from -0.4 to 0.4.

a

	I-I	I-G	I-N
Fish	3	18	7
Mixed	3	0	4
Unknown	2	7	9

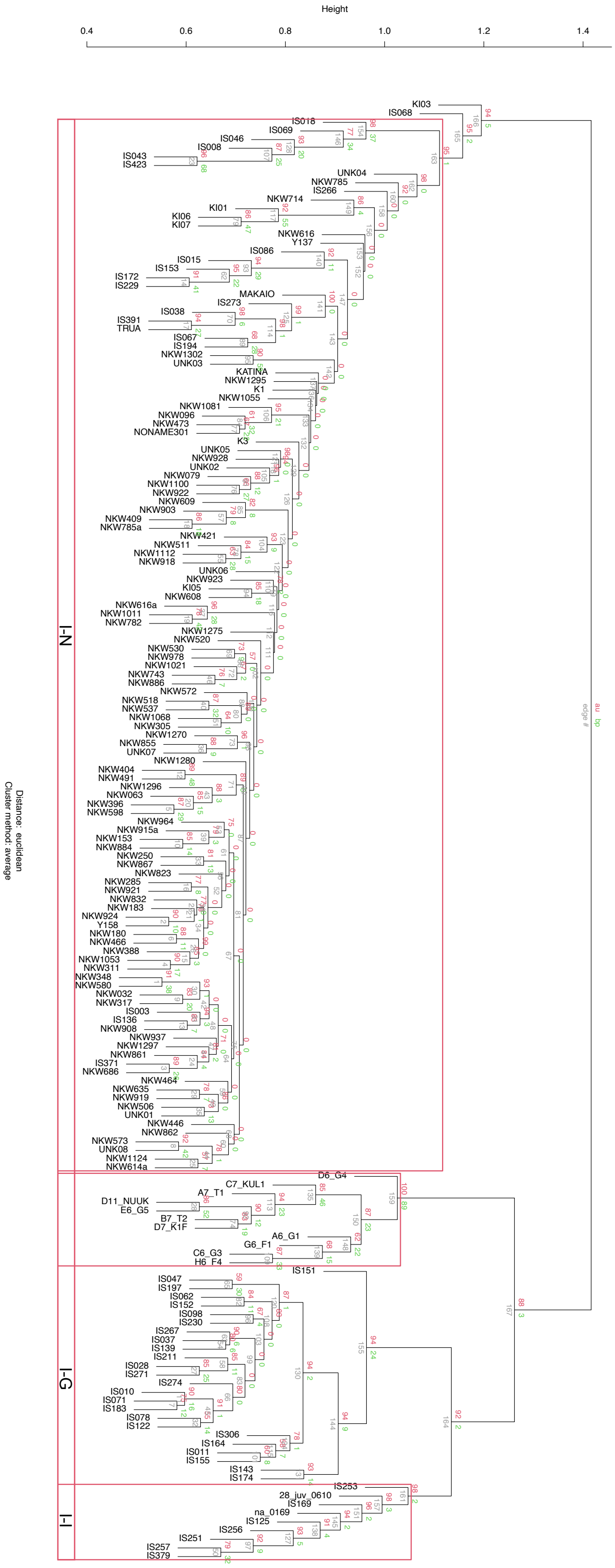

b

	western	eastern
Fish	18	9
Mixed	0	8
Unknown	7	12

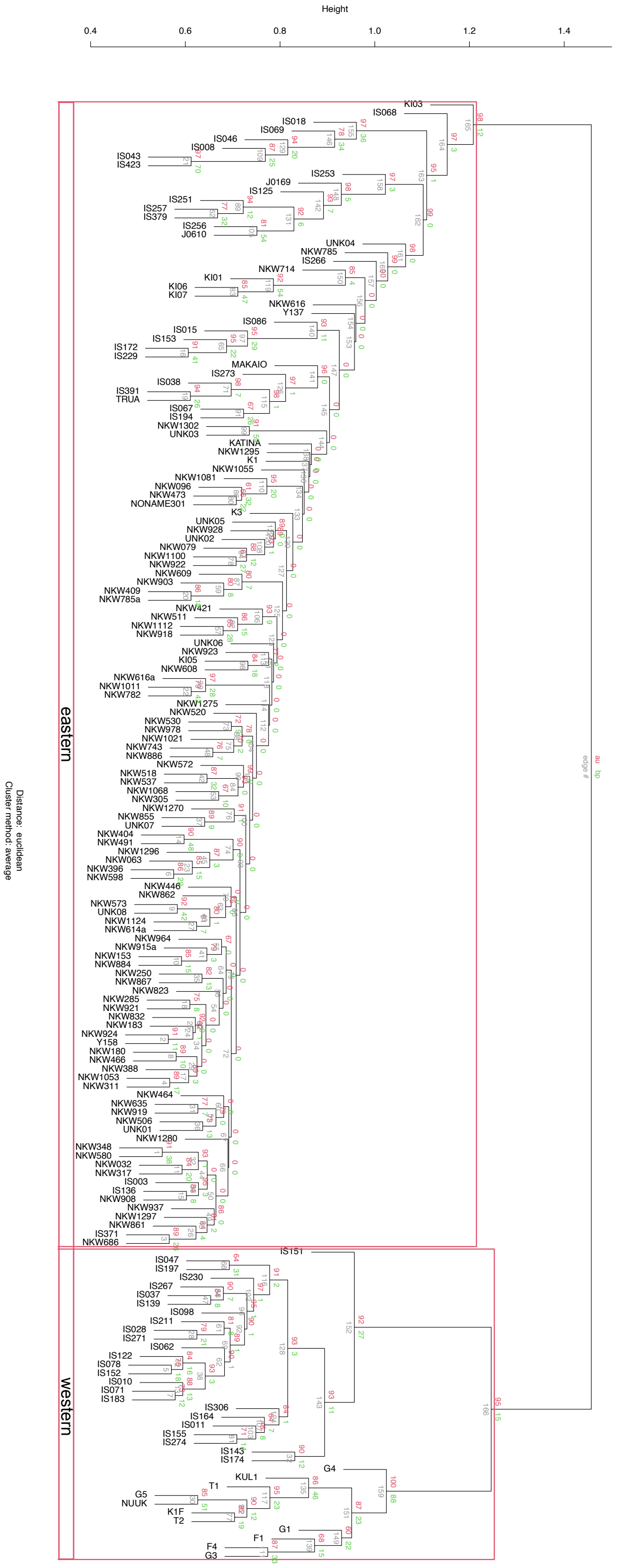

Fig. S5. Relatedness-based hierarchical clustering and association between diet types and cluster assignment of Icelandic killer whales. A distance matrix was calculated using 1− rxy as the distance metric, and hierarchical clustering was performed using the pvclust package (version 2.2-0, Suzuki and Shimodaira, 2006). Clusters with strong support (AU p-value > 0.95) are highlighted as red rectangles. The tables contain counts of Icelandic individuals with their respective diet types found in each cluster - rooted in the deeper nodes of the dendrogram. **a** Hierarchical clustering was performed with the entire data set (data set 4, n=169). The table contains the counts of Icelandic individuals with their respective diet types found in the three (out of four) clusters that contain Icelandic individuals. I-I refers to the cluster at the left extreme of the dendrogram, I-G refers to the Icelandic cluster that exhibits recent gene flow with Greenland and I-N refers to the Icelandic individuals clustering with Norwegian individuals. A Fisher's exact test revealed a statistically significant association between diet types and cluster assignment of Icelandic killer whales $p = 0.00964$. **b** Hierarchical clustering was performed on the remaining data set after IS169 was removed (n=168). The table contains the counts of Icelandic individuals with their respective diet types found in two clusters. The western cluster contained Icelandic and Greenlandic individuals, and the eastern cluster contained Icelandic and Norwegian individuals. A Fisher's exact test revealed a statistically significant association between diet types and cluster assignment of Icelandic killer whales $p=0.001702$.

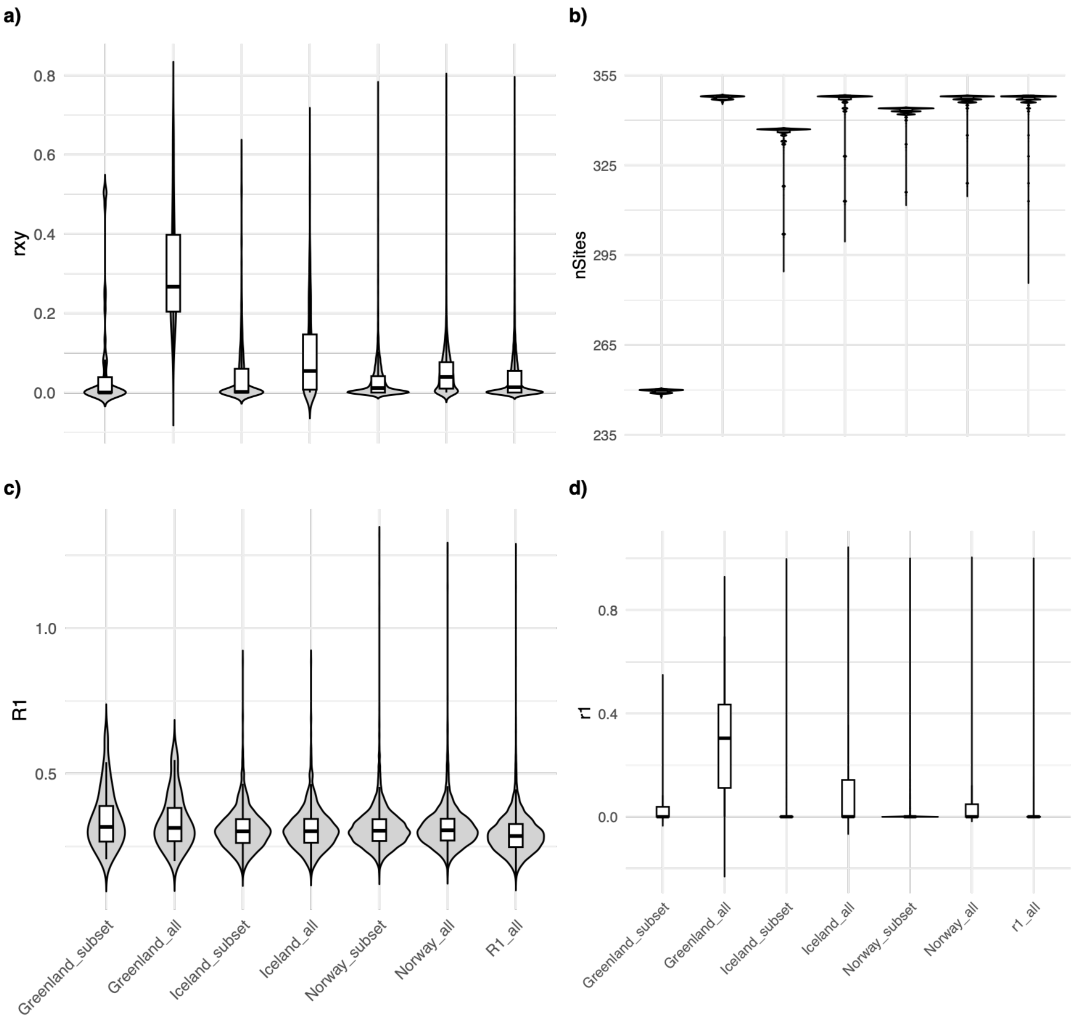

Fig. S6. Evaluating the effect of background relatedness. a,c,d Distribution of r_{xy} (Hedrick and Lacy 2015), R1 (Waples et al. 2019), r_1 (Jacquard, 1972), respectively, for the same pairs estimated within each metapopulation deme and across the entire metapopulation. **b** Number of polymorphic sites within each metapopulation deme and across the entire metapopulation.

3. Command lines

```
#### mapping
### Data preparation
# mitochondrial reference genome
# NC_023889.1 Orcinus orca isolate ENAHN1 mitochondrion, complete genome
(https://www.ncbi.nlm.nih.gov/nuccore/NC_023889.1?report=fasta)

# nuclear reference genome
wget
https://ftp.ncbi.nlm.nih.gov/genomes/all/GCA/000/331/955/GCA_000331955.2_Oorc_1.1/G
CA_000331955.2_Oorc_1.1_genomic.fna.gz

# sequencing data received, as e.g. for individual IS391
A10_IS391_5265_EKDL220015932-1A_H5GYMDSX5_L4_1.fq.gz
A10_IS391_5265_EKDL220015932-1A_H5GYMDSX5_L4_2.fq.gz

### Trim adapters
module load gcc/4.8.2 gdc adapterremoval/2.1.7
 ./path/adapterremoval/adapterremoval-2.3.1/build/AdapterRemoval --file1 name_1.fq.gz --
file2 name_2.fq.gz --basename name --minlength 50 --trimns --collapse --gzip
# e.g. for individual IS391
~/adapterremoval-2.3.1/build/AdapterRemoval --file1 A10_IS391_5265_EKDL220015932-
1A_H5GYMDSX5_L4_1.fq.gz --file2  A10_IS391_5265_EKDL220015932-
1A_H5GYMDSX5_L4_2.fq.gz --basename A10_IS391_5265 --minlength 50 --trimns --
collapse --gzip

# output, e.g. for individual IS391
#A10_IS391_5265.pair1.truncated.gz
#A10_IS391_5265.pair2.truncated.gz
#A10_IS391_5265.collapsed.gz
#A10_IS391_5265.collapsed.truncated.gz
#A10_IS391_5265.singleton.truncated.gz #removed for further mapping steps
#A10_IS391_5265.discarded.gz #removed for further mapping steps
#A10_IS391_5265.settings #removed for further mapping steps

### map the data to the reference genome, using BWA and scaffold for SNPs
module load bwa/0.7.15
./path/bwa/bwa mem -t 4 path/unplaced.scaf.fna name.pair1.truncated.gz
name.pair2.truncated.gz | gzip -3 > name_paired_read.sam.gz
# e.g. for individual IS391
bwa mem -t 4 ~/data/refgenome/unplaced.scaf.fna A10_IS391_5265.pair1.truncated.gz
A10_IS391_5265.pair2.truncated.gz | gzip -3 > A10_IS391_5265_paired_read.sam.gz
bwa mem -t 4 ~/data/refgenome/unplaced.scaf.fna A10_IS391_5265.collapsed.gz | gzip -3 >
A10_IS391_5265.collapsed.sam.gz
bwa mem -t 4 ~/data/refgenome/unplaced.scaf.fna A10_IS391_5265.collapsed.truncated.gz |
gzip -3 > A10_IS391_5265.collapsed.truncated.sam.gz

### merge .sam files for the same sample using samtools
module load samtools/1.12
```

```
samtools merge name_merged.sam.gz name_paired_read.sam.gz name_collapsed.sam.gz
name_collapsed_truncated.sam.gz
# e.g. for individual IS391
samtools merge A10_IS391_5265_merged.sam.gz A10_IS391_5265_paired_read.sam.gz
A10_IS391_5265.collapsed.sam.gz A10_IS391_5265.collapsed.truncated.sam.gz

### convert .sam to .bam file using samtools
samtools view -bSh name_merged.sam.gz | samtools sort -o name.bam
# e.g. for individual IS391
samtools view -bSh A10_IS391_5265_merged.sam.gz | samtools sort -o
A10_IS391_5265_merged.bam

### collapse clonal reads using rmdup function of samtools
samtools rmdup -s name.bam name_rmdup.bam
# e.g. for individual IS391
samtools rmdup -s A10_IS391_5265_merged.bam A10_IS391_5265_merged_rmdup.bam

### rename to final .bam file
# e.g. for individual IS391
mv A10_IS391_5265_merged_rmdup.bam IS391.bam

### make a .bamlist
echo 'IS391.bam
[...]' -> nuDNA_n171.bamlist

### Depth of coverage with nuDNA_n171.bamlist (n=171), using angsd
module load angsd/0.925
angsd -bam nuDNA_n171.bamlist -sites ~/data/refgenome/coordinates.sites -doDepth 1 -out
nuDNA_n171 -doCounts 1 -nInd 171


#### nuDNA population-based methods
### Extract genotype likelihoods in .beagle format, with nuDNA_n171.bamlist (n=171),
using angsd
module load angsd/0.925
angsd -bam nuDNA_n171.bamlist -sites ~/data/refgenome/coordinates.sites -GL 1 -doGlf 2 -
doMajorMinor 1 -SNP_pval 1e-6 -doMaf 1 -out nuDNA_n171.gL -minMapQ 30 -minQ 30 -
uniqueonly 1 -remove_bads 1 -minInd 25 -C 50 -baq 1 -ref
~/data/refgenome/unplaced.scaf.fna

### covariance matrix for PCA and NGSadmix, using pcangsd in python
module load gcc/4.8.2 gdc python/3.6.1 pcangsd/0.98
pcangsd.py -beagle nuDNA_n171.gL.beagle.gz -o nuDNA_n171 -threads 4
python -c "import numpy as np; cov_matrix = np.load('nuDNA_n171.cov.npy');
np.savetxt('nuDNA_n171.cov', cov_matrix, fmt='%0.10f')"

### PCAs (colour-coded re location and diet respectively)
# Load necessary libraries
library(ggplot2)
library(dplyr)
```

```
library(tidyverse)
library(patchwork)

# Read covariance matrix from the .cov file
cov_matrix <- as.matrix(read.table("~/path/cov_nuDNA.cov"))

# Calculate eigenvalues and eigenvectors and PC scores
eigen_values <- eigen(cov_matrix)
scores <- eigen_values$vectors %*% diag(sqrt(eigen_values$values))

# Calculate the percentage of variance explained by each PC
variance_explained <- eigen_values$values / sum(eigen_values$values) * 100

# Prepare the data for plotting
pca_df <- data.frame(PC1 = scores[, 1], PC2 = scores[, 2])

# Read the CSV file with default names and actual IDs names
labels_data <- read.csv("~/path/relabel_pca.csv")

# Ensure pca_df has row names to replace
if(is.null(rownames(pca_df))) {
  rownames(pca_df) <- as.character(1:nrow(pca_df))
}

# Create a named vector from labels_data
label_map <- setNames(labels_data$new, labels_data$old)

# Update row names in pca_df
rownames(pca_df) <- label_map[rownames(pca_df)]

# Convert row names to a column for plotting
pca_df$Sample <- rownames(pca_df)

# Merge pca_df with labels_data to get location information
pca_df <- merge(pca_df, labels_data, by.x = "Sample", by.y = "new")

# Generate the plot with adjusted label positions and fixed aspect ratio for Sampling Location
p <- ggplot(pca_df, aes(x = PC1, y = PC2, fill = location)) +
  geom_point(shape = 21, size = 2, color = "NA") +
  theme_minimal() +
  scale_fill_manual(name = "Sampling Location", values = c("Iceland" = "#FFB800",
"Greenland" = "indianred1", "Norway" = "#56B4E9")) +
  labs(title = "",
       x = paste("PC 1 (", sprintf("%.2f%%", variance_explained[1]), ")", sep = ""),
       y = paste("PC 2 (", sprintf("%.2f%%", variance_explained[2]), ")", sep = "")) +
  guides(fill = guide_legend(title = "Sampling Location")) +
  coord_fixed(ratio = 1)

# Add colored ellipses around the data points for each group (sampling location)
pca1_plot <- p +
```

```
  stat_ellipse(aes(group = location, color = location, fill = location),
              level = 0.95,
              geom = "polygon",
              alpha = 0.1,
              linetype = 0) +
  scale_color_manual(name = "Sampling Location",
                  values = c("Iceland" = "#FFB800", "Greenland" = "indianred1", "Norway" =
"#56B4E9")) +
  guides(color = guide_legend(title = "Sampling Location")) +
  scale_x_continuous(limits = c(-1.2, 0.7), breaks = seq(-1.0, 0.5, by = 0.5)) +
  scale_y_continuous(limits = c(-0.5, 0.7), breaks = seq(-0.3, 0.6, by = 0.3))

# Print the first plot (PCA1)
print(pca1_plot)

# Extract axis limits from the first plot
x_limits <- ggplot_build(pca1_plot)$layout$panel_params[[1]]$x.range
y_limits <- ggplot_build(pca1_plot)$layout$panel_params[[1]]$y.range

# PCA 2: Plot colored by diet
# Read the CSV file with diet data
diet_data <- read.csv("~/path/diet.csv")

# Filter and merge diet data
filtered_diet_data <- diet_data %>%
  filter(ID %in% labels_data$new) %>%
  distinct(ID, .keep_all = TRUE)

merged_data <- labels_data %>%
  left_join(filtered_diet_data %>% select(ID, Diet, Movement), by = c("new" = "ID")) %>%
  rename(Ecology = Diet)

merged_data$Ecology <- trimws(merged_data$Ecology)

# Merge PCA data with merged_data for diet plot
pca_df_diet <- merge(pca_df, merged_data, by.x = "Sample", by.y = "new")

# PCA 2: Plot colored by diet
# Generate the plot with adjusted label positions and fixed aspect ratio for Diet
pca2_plot <- ggplot(pca_df_diet, aes(x = PC1, y = PC2, fill = Ecology)) +
  geom_point(shape = 21, size = 2, color = "NA") +
  theme_minimal() +
  scale_fill_manual(name = "Diet", values = c("Unknown" = "grey", "Fish" = "#56B4E9",
"Mixed" = "indianred1")) +
  labs(title = "",
       x = paste("PC 1 (", sprintf("%.2f%%", variance_explained[1]), ")", sep = ""),
       y = paste("PC 2 (", sprintf("%.2f%%", variance_explained[2]), ")", sep = "")) +
  guides(fill = guide_legend(title = "Diet")) +
  coord_fixed(ratio = 1) +
  # Set axis limits and breaks using scale_x_continuous and scale_y_continuous
```

```
  scale_x_continuous(limits = c(-1.2, 0.7), breaks = seq(-1.0, 0.5, by = 0.5)) +
  scale_y_continuous(limits = c(-0.5, 0.7), breaks = seq(-0.3, 0.6, by = 0.3))
pca2_plot

# Combine the two plots side by side using patchwork
combined_plot <- pca1_plot + pca2_plot + plot_layout(ncol = 2)

# Display the combined plot
print(combined_plot)

### PCA-eigenvalues plot
# Prepare the data for plotting the variance explained
variance_df <- data.frame(PC = paste0("PC", 1:length(variance_explained)),
                          Variance = variance_explained)

# Sort the variance_df by Variance in descending order and ensure PC is a factor
variance_df <- variance_df %>%
  arrange(desc(Variance))
variance_df$PC <- factor(variance_df$PC, levels = variance_df$PC)

# Plot the eigenvalues (variance explained)
eigen_plot <- ggplot(variance_df, aes(x = PC, y = Variance)) +
  geom_bar(stat = "identity") +
  theme_minimal() +
  labs(title = "",
       x = "Principal Component",
       y = "Variance Explained (%)") +
  theme(axis.text.x = element_blank(),
        axis.ticks.x = element_blank(),
        panel.grid.major = element_blank(),
        panel.grid.minor = element_blank(),
        plot.title = element_blank())

# Show the eigenvalues plot
print(eigen_plot)

### ngsadmix
# Define base command with the full path to NGSadmix
BASE_CMD="/path/NGSadmix"

# Define the range for K and the seeds
for K in {1..10}; do
    for run in {1..5}; do
        # Set different seeds for each run
        if [ $run -eq 1 ]; then SEED=250000; fi
        if [ $run -eq 2 ]; then SEED=500000; fi
        if [ $run -eq 3 ]; then SEED=750000; fi
        if [ $run -eq 4 ]; then SEED=1000000; fi
        if [ $run -eq 5 ]; then SEED=1250000; fi
```

```
        # Set output file name
        OUTPUT_FILE="Oct_2024_IGLN_K${K}.${run}"

        # Run the command
        echo "Running NGSadmix for K=${K}, run=${run} with seed=${SEED}..."
        $BASE_CMD -likes all_OctIGLN.gL.beagle.gz -seed $SEED -K $K -o
$OUTPUT_FILE -P 10
    done
done

### evaladmix
## entire data set
~/evalAdmix/evalAdmix -beagle all_OctIGLN.gL.beagle.gz -fname ngsadmix_K2.3.fopt.gz -
qname ngsadmix_K2.3.qopt -P 10 -o evalout240321.corres.txt

# in R for all samples
source("~/path/visFuns.R")

# read population labels and estimated admixture proportions
pop <- read.table("~/path/ID_location.txt", header=FALSE)
q <- read.table("~/path/ngsadmix_K2.3.qopt", quote="\"", comment.char="")

# order according to population and plot the NGSadmix reults
ord <- orderInds(pop = as.vector(pop[,2]), q = q)
barplot(t(q)[,ord],col = c("#56B4E9",
"indianred1"),space=0,border=NA,xlab="Individuals",ylab="")
text(sort(tapply(1:nrow(pop),pop[ord,1],mean)),-0.05,unique(pop[ord,1]),xpd=T, srt = 90,
cex = 0.5)
#abline(v=cumsum(sapply(unique(pop[ord,1]),function(x){sum(pop[ord,1]==x)})),col=1,lwd
=1.2)

r <- read.table("~/path/evalout.corres.txt", header=FALSE)

# Plot correlation of residuals
plotCorRes(cor_mat = r, pop = as.vector(pop[,2]), ord = ord, title="upper triangle: indiviudal
pairwise correlation of residuals, lower: population mean", cex.lab=0.7, cex.lab.2 = 0.7,
        cex.main=0.5, adjlab = 0.1, rotatelabpop = 90, rotatelabsuperpop = 0, lineswidth = 1,
lineswidthsuperpop = 2,
        adjlabsuperpop = 0.16, max_z=0.1, min_z=-0.1)


### subset with homogeneous ancestry (ngsadmix and evaladmix)
#extract subset with homogeneous ancestry from file with genotype likelihoods
#save as tab-delimited.txt and rename .beagle
#in terminal
gzip evaladmix_subset.beagle
evaladmix_subset.beagle.gz

#ngsadmix k=2
```

```
~/NGSadmix -likes evaladmix_subset.beagle.gz -seed 500000 -K 2 -o ngsadmix_K2.subset -
P 10

#using subset beagle
~/evalAdmix/evalAdmix -beagle evaladmix_subset.beagle.gz -fname
ngsadmix_K2.subset.fopt.gz -qname ngsadmix_K2.subset.qopt -o
eval_subset240325.corres.txt


#### nuDNA kinship-based methods
### Use ngsRelate to estimate pairwise relatedness
# First generate a file with allele frequencies (angsdput.mafs.gz) and a file with genotype
likelihoods (angsdput.glf.gz), using angsd
module load angsd/0.925
angsd -b nuDNA_n169.bamlist -sites ~/data/refgenome/coordinates.sites -gl 2 -domajorminor
1 -snp_pval 1e-6 -domaf 1 -minmaf 0.05 -doGlf 3

# Then extract the frequency column from the allele frequency file and remove the header (to
make it in the format NgsRelate needs), using angsd
zcat angsdput.mafs.gz | cut -f5 |sed 1d >freq

# run ngsRelate
module load gcc/4.8.2 gdc ngsrelate/0.1
~/ngsRelate/ngsRelate -g angsdput.glf.gz -n 169 -f freq  -O nuDNA__n169_rxy

### rxy bootstrapping and heatmap in R
# Load necessary libraries
library(pvclust)
library(ggplot2)
library(ggdendro)
library(reshape2)
library(heatmaply)
library(pheatmap)

# Load data
relatedness_all <- read.delim("~/path/rxy_n169_renamed.txt", header=T)

# Reduce table to x,y,rxy
relatedness_rxy <- relatedness_all[, c("x", "y", "rxy")]

# Symmetrise the data
# Add the reverse pair (y, x) with the same relatedness value (rxy)
relatedness_rxy_symm <- rbind(relatedness_rxy,
                              data.frame(x = relatedness_rxy$y,
                                         y = relatedness_rxy$x,
                                         rxy = relatedness_rxy$rxy))

# convert to wide format matrix
relatedness_rxy_wide <- reshape2::acast(relatedness_rxy_symm, x ~ y, value.var = "rxy", fill
= 0)
```

```
# create distance matrix
# Create a distance matrix using relatedness values (1 - rxy) for clustering
dist_matrix <- as.dist(1 - relatedness_rxy_wide)

# hierarchical clustering with pvclust using bootstrap resampling
set.seed(123)
pv_clustering <- pvclust(as.matrix(relatedness_rxy_wide), method.hclust = "average",
method.dist = "euclidean", nboot = 10000)

# Plot the dendrogram with AU and BP p-values
plot(pv_clustering, main = "Dendrogram with Significant Clusters (AU p-value > 0.95)")

# Highlight clusters with strong support (AU p-value > 0.95)
pvrect(pv_clustering, alpha = 0.95, pv = "au", border = 2, lwd = 2)

# Create a black and white color palette for the heatmap
bw_palette <- colorRampPalette(c("white", "black"))(100)

# Static bw heatmap using pheatmap
pheatmap::pheatmap(
  relatedness_rxy_wide,
  cluster_rows = pv_clustering$hclust,
  cluster_cols = pv_clustering$hclust,
  color = bw_palette,
  main = "",
  scale = "none",
  border_color = NA,
  treeheight_row = 50,
  treeheight_col = 50
)

# Extract AU (approximately unbiased) and BP (bootstrap probability) p-values for each
cluster
au_pvalues <- pv_clustering$edges[, "au"]
bp_pvalues <- pv_clustering$edges[, "bp"]

# Create a data frame to store the p-values for each cluster
pvalues_df <- data.frame(
  cluster = 1:length(au_pvalues),
  AU_pvalue = au_pvalues,
  BP_pvalue = bp_pvalues
)

# Print p-value results
print(pvalues_df)

# Total number of clusters
total_clusters <- length(au_pvalues)
```

```
# Number of significant clusters (AU p-value > 0.95)
strong_clusters <- sum(au_pvalues > 0.95)

cat("Total number of clusters:", total_clusters, "\n")
cat("Number of clusters with AU p-values > 0.95:", strong_clusters, "\n")

# extract cluster members from pvclust results
get_cluster_members <- function(clustering_result, cluster_idx) {
  cluster_members <- cutree(clustering_result$hclust, k = cluster_idx)
  return(cluster_members)
}

# Get significant clusters (AU p-value > 0.95)
significant_clusters <- which(au_pvalues > 0.95)

# Initialise a counter for dyads
dyad_count <- 0

# Loop through significant clusters and count how many are dyads
for (cluster_idx in significant_clusters) {
  # Get the members of the cluster
  cluster_members <- get_cluster_members(pv_clustering, cluster_idx)

  # Check if the cluster is a dyad (contains exactly two members)
  if (length(unique(cluster_members)) == 2) {
    dyad_count <- dyad_count + 1
  }
}

# Output the total number of dyads among the significant clusters
cat("Number of significant clusters that are dyads:", dyad_count, "\n")

### Fisher's Exact Test for Diet vs Icelandic Cluster
# Load necessary libraries
library(dplyr)

# Load data
diet_mtDNA <- read.csv("~/path/diet_ mtDNA.csv")
cluster_data <- read.csv("~/path/rxy_cluster_wIS169.csv")

# Merge the two datasets on the "ID" column
merged_data <- merge(diet_mtDNA, cluster_data, by = "ID")
diet_cluster_table <- table(merged_data$Diet, merged_data$cluster)

# Perform Fisher's Exact Test for Diet vs Cluster
diet_fisher_result <- fisher.test(diet_cluster_table)

# Print the results of Fisher's Exact Test for Diet vs Cluster
print(diet_fisher_result)
```

```
### infer PO
##extract known-MO in R
# Load necessary library
library(dplyr)

# Load relatedness data
relatedness_all <- read.delim("~/path/rxy_n169_renamed.txt", header = TRUE)

# Define the known mother-offspring pairs as a data frame
known_pairs <- data.frame(
  a = c("G1", "G4", "KUL1", "J0610", "IS253", "KATINA", "IS153", "NKW785a", "KI03",
"K1", "NKW785", "NKW616a", "KI03", "KI01"),
  b = c("F1", "F4", "K1F", "IS253", "IS256", "MAKAIO", "IS086", "NKW785", "KI07",
"K3", "NKW903", "NKW616", "KI06", "KI03")
)

# Filter the relatedness_all data for the specified pairs
filtered_data <- relatedness_all %>%
  filter(
    (a %in% known_pairs$a & b %in% known_pairs$b & a == known_pairs[match(b,
known_pairs$b), "a"]) |
      (b %in% known_pairs$a & a %in% known_pairs$b & b == known_pairs[match(a,
known_pairs$b), "a"])
  )

# View the extracted table to confirm the correct pairs are included
print(filtered_data)

## stats
# Load necessary libraries
library(dplyr)

# Check available columns
print(colnames(relatedness_all))

# Filter data for exact pairs and select required columns
filtered_data <- relatedness_all %>%
  filter(
    (a %in% known_pairs$a & b %in% known_pairs$b & a == known_pairs[match(b,
known_pairs$b), "a"]) |
      (b %in% known_pairs$a & a %in% known_pairs$b & b == known_pairs[match(a,
known_pairs$b), "a"])
  ) %>%
  select(a, b, J9, J8, J7, rxy, theta, R0, R1, KING)

# Print filtered data to confirm
print(filtered_data)

# Calculate statistics
statistics <- filtered_data %>%
```

```
  summarise(across(c(J9, J8, J7, rxy, theta, R0, R1, KING),
                  list(median = ~median(.x, na.rm = TRUE),
                       se = ~sd(.x, na.rm = TRUE) / sqrt(n()),
                       min = ~min(.x, na.rm = TRUE),
                       max = ~max(.x, na.rm = TRUE))))

# Print the extended statistics
print(statistics)

#filter data according to MO statistics
library(dplyr)
library(tidyr)

# Load the minimum and maximum thresholds data and map names
minmax <- read.delim("~/path/MO_statistics_table.txt", header = TRUE) %>%
  rename(estimator = X) %>%
  mutate(estimator = case_when(
    estimator == "r0" ~ "J9",
    estimator == "r1" ~ "J8",
    estimator == "r2" ~ "J7",
    estimator == "rxy" ~ "rxy",
    TRUE ~ estimator
  )) %>%
  select(estimator, min, max)

# Print to confirm the renamed columns
print(minmax)

# Function to apply the min and max filters
filter_data <- function(data, stats) {
  results <- data

  # Apply filters for each row in stats
  for (i in 1:nrow(stats)) {
    estimator <- stats$estimator[i]
    min_val <- stats$min[i]
    max_val <- stats$max[i]

    # Dynamically filter data within min and max range for the current estimator
    results <- results %>%
      filter(between(.data[[estimator]], min_val, max_val))
  }

  return(results)
}

# Apply filtering function
filtered_results <- filter_data(relatedness_all, minmax)

# Select necessary columns from the filtered results
```

```
final_filtered_data <- filtered_results %>%
  select(a, b, J9, J8, J7, rxy, theta, R0, R1, KING)

# Print the final filtered table
print(final_filtered_data)


## R0-R1 and R1-KING scatterplots, with known and inferred P-O pairs colour-coded in red
# Load necessary libraries
library(ggplot2)
library(dplyr)
library(patchwork)

# Load relatedness data
relatedness_all <- read.delim("~/path/rxy_n169_renamed.txt", header = TRUE)

# Default color to grey
relatedness_all$color <- "grey"

# Define specific pairs to color as 'indianred1'
specific_pairs <- data.frame(
  a = c("IS172", "IS153", "IS069", "IS069", "IS253", "IS253", "IS152", "J0610", "IS038",
"IS230", "G1", "KUL1", "G4", "MAKAIO", "K1", "KI01", "KI03", "KI03", "NKW1055",
"NKW616", "NKW714", "NKW785", "NKW785"),
  b = c("IS086", "IS086", "IS046", "IS008", "J0610", "IS256", "IS169", "IS169", "IS266",
"IS125", "F1", "K1F", "F4", "KATINA", "K3", "KI03", "KI06", "KI07", "NKW1295",
"NKW616a", "Y137", "NKW785a", "NKW903")
)

# Interaction term to uniquely identify pairs
specific_pairs$pair_id <- with(specific_pairs, paste(pmin(a, b), pmax(a, b), sep = "_"))
relatedness_all$pair_id <- with(relatedness_all, paste(pmin(a, b), pmax(a, b), sep = "_"))

# Assign colors based on these specific pairs
relatedness_all$color[relatedness_all$pair_id %in% specific_pairs$pair_id] <- "indianred1"

# Set factor levels to change the legend order
relatedness_all$color <- factor(relatedness_all$color, levels = c("indianred1", "grey"))

# Define a function to create plots
create_plot <- function(x, y, xlabel, ylabel, show_legend) {
  plot <- ggplot(relatedness_all, aes_string(x = x, y = y, color = "color")) +
    geom_point(size = 0.7) +
    scale_color_manual(values = c("grey" = "grey", "indianred1" = "indianred1"),
                       labels = c("Parent-Offspring", "Other")) +
    labs(x = xlabel, y = ylabel) +
    theme_minimal() +
    theme(legend.position = if (show_legend) c(0.95, 0.95) else "none",
          legend.justification = c("right", "top"),
          legend.background = element_rect(fill = "white", color = NA)) +
```

```
    guides(color = guide_legend(title = NULL))
  return(plot)
}

# Create the upper plot for R0 vs. R1 (with legend)
plot_upper <- create_plot("R1", "R0", "R1", "R0", show_legend = TRUE)

# Create the lower plot for KING vs. R1 (without legend)
plot_lower <- create_plot("R1", "KING", "R1", "KING", show_legend = FALSE)

# Combine the plots and add a common legend
combined_plot <- plot_upper / plot_lower
print(combined_plot)


#### mtDNA
### mtDNA alignement in Seaview
# convert .bam to .fasta
angsd -i IS391.bam -doFasta 2 -doCounts 1 -out IS391
# import to Seaview
# visually inspect for misalignement
# visually inspect for SNPs
# export alignement as .fasta file

### Visualise SNPs in SNPIT, using python
module load gdc python/3.6.1

snipit aligned_mtseq.fasta \
  -r Ref \
  --include-positions 1845 3173 6413 8466 11868 15132 15549 15792 16164 \
  -o 2024-03-27SNPIT_all \
  -s \
  -f pdf \
  --height 35 \
  --width 15 \
  -c classic \
  --sort-by-mutation-number

### use popart and genodive softwares
```

References

Enk JM, Devault AM, Kuch M, Murgha YE, Rouillard JM, Poinar HN (2014) Ancient whole genome enrichment using baits built from modern DNA. Mol Biol Evol 31(5):1292–1294

Excoffier L, Smouse PE, Quattro JM (1992) Analysis of molecular variance inferred from metric distances among DNA haplotypes - application to human mitochondrial-DNA restriction data. Genetics 131:479-491

Foote AD and Morin PA (2016) Genome-wide SNP data suggest complex ancestry of sympatric North Pacific killer whale ecotypes. Heredity 117(5):316-325

Hedrick PW and Lacy RC (2015) Measuring relatedness between inbred individuals. J Hered 106(1):20–25

Jacquard A (1972) Genetic information given by a relative. Biometrics 28(4):1101–1114

Jourdain E, Karoliussen R, Fordyce MS, Langangen Ø, Robeck T, Borgå K et al. (2024) Social and genetic connectivity despite ecological variation in a killer whale network. Proc R Soc B (291):e20240524

Lee WC (2003) Testing the genetic relation between two individuals using a panel of frequency-unknown single nucleotide polymorphisms. Ann Hum Genet (67):618–619

Manichaikul A, Mychaleckyj JC, Rich SS, Daly K, Sale M, Chen W-M (2010) Robust relationship inference in genome-wide association studies. Bioinformatics 26: 2867–2873

Meirmans PG (2020) GENODIVE version 3.0: Easy-to-use software for the analysis of genetic data of diploids and polyploids, Mol Ecol Resour 20: 1126-1131

Michalakis Y and Excoffier L. 1996. A generic estimation of population subdivision using distances between alleles with special reference for microsatellite loci. Genetics 142:1061-1064

Suzuki R, Shimodaira H (2006) Pvclust: an R package for assessing the uncertainty in hierarchical clustering. Bioinformatics 22(12):1540–1542

Moura AE, Kenny JG, Chaudhuri R, Hughes MA, Welch AJ, Reisinger RR et al. (2014) Population genomics of the killer whale indicates ecotype evolution in sympatry involving both selection and drift. Mol Ecol 23(21):5179–5192

Tavares SB, Samarra FIP, Pascoal S, Graves JA, Miller PJO (2018) Killer whales (Orcinus orca) in Iceland show weak genetic structure among diverse isotopic signatures and observed movement patterns. Ecol Evol 8(23):11900–11913

Waples RK, Albrechtsen A, Moltke I (2019) Allele frequency-free inference of close familial relationships from genotypes or low-depth sequencing data. Mol Ecol 28(1):35-48
